# Supplementary material for: The impact of reproductive factors on the metabolic profile of females from menarche to menopause
Source: Nat Commun. 2024 Feb 6;15:1103. doi: 10.1038/s41467-023-44459-6 (PMC10847109; doi:10.1038/s41467-023-44459-6)

**Supplementary figures**

Suppl Fig 1. Multivariable regression (red) and Mendelian randomization (black) estimates for the relation between older age at menarche and metabolic measures among females.

Suppl Fig 2. Multivariable regression estimates for the relation between older age at menarche and metabolic measures among females (comparing different model adjustments)

Suppl Fig 3. Multivariable regression estimates for the relation between age at menarche (<13 years, 13-14 years, >14 years) and metabolic measures among females

Suppl Fig 4. Multivariable regression estimates for the relation between age at menarche (restricted cubic splines with knots placed at ages 11, 13, and 15) and metabolic measures among females

Suppl Fig 5. Univariable and multivariable Mendelian randomization estimates for the relation between older age at menarche and NMR metabolomics measures among females

Suppl Fig 6. Univariable and multivariable Mendelian randomization estimates for the relation between older age at menarche and clinical chemistry biomarkers among females

Suppl Fig 7. Multivariable regression (red) and Mendelian randomization (black) estimates for the relation between higher parity and metabolic measures among females.

Suppl Fig 8. Multivariable regression estimates for the relation between higher parity and metabolic measures among females (comparing different model adjustments)

Suppl Fig 9. Multivariable regression estimates for the relation between parity (0, 1, 2, 3+) and metabolic measures among females

Suppl Fig 10. Multivariable regression estimates for the relation between parity (restricted cubic splines with knots placed at 1 2 and 3 births) and metabolic measures among females

Suppl Fig 11 Multivariable regression (red) and Mendelian randomization (black) estimates for the relation between older age at natural menopause and metabolic measures among females

Suppl Fig 12. Multivariable regression estimates for the relation between older age at natural menopause and metabolic measures among females (comparing different model adjustments)

Suppl Fig 13. Multivariable regression estimates for the relation between age at menopause (<49, 49-50, 51-53, >53) and metabolic measures among females

Suppl Fig 14. Multivariable regression estimates for the relation between age at menopause (restricted cubic splines with knots placed at ages 40, 49, 52, and 56) and metabolic measures among females

Suppl Fig 15. Age-combined and age-stratified estimates for the association between age at natural menarche and clinical chemistry biomarkers using multivariable regression (red) and Mendelian randomization (blue) restricted to women with data on age at menopause or Mendelian randomization (black) using data from all women.

Suppl Fig 16A. Age-combined and age-stratified estimates for the association between age at natural menarche and clinical chemistry biomarkers excluding users of statins at baseline estimated using multivariable regression (red) and Mendelian randomization (blue) restricted to women with data on age at menopause or Mendelian randomization (black) using data from all women.

Suppl Fig 16B. Age-combined and age-stratified estimates for the association between age at natural menarche and clinical chemistry biomarkers excluding users of hormone replacement therapy (HRT) at baseline estimated using multivariable regression (red) and Mendelian randomization (blue) restricted to women with data on age at menopause or Mendelian randomization (black) using data from all women.

Suppl Fig 17. Mendelian randomization estimates for the relation between reproductive markers and conventional biomarkers among unrelated individuals and within siblings.

Suppl Fig 18. Mendelian randomization estimates for the relation between older age at menarche and metabolic measures among females (comparing different Mendelian randomization methods)

Suppl Fig 19. Mendelian randomization estimates for the relation between higher parity and metabolic measures among females (comparing different Mendelian randomization methods)

Suppl Fig 20. Mendelian randomization estimates for the relation between older age at natural menopause and metabolic measures among females (compare different Mendelian randomization methods)

Suppl Fig 21. Mendelian randomization estimates for the relation between older age at menarche and metabolic measures among females (comparing different SNP sets)

Suppl Fig 22. Mendelian randomization estimates for the relation between older age at natural menopause and metabolic measures among females (comparing different SNP sets)

# Suppl Fig 1. Multivariable regression (red) and Mendelian randomization (black) estimates for the relation between older age at menarche and metabolic measures among females. Footnote: MV=multivariable; MR=Mendelian Randomisation. Results are mean differences presented as standard unit changes in metabolic measure per 1 year increase in age at menarche. Circles denote the mean differences and indicate p-value < 0.00093 (filled circles) or ≥ 0.00093 (hollow circles). Horizontal bars denote 95% confidence intervals. Multivariable regression models (ordinary least squares, two-sided regression coefficients reported) were adjusted for age at recruitment, body size at age 10 and education (N=61,920). Mendelian randomization models were estimated using the inverse variance weighted method (N= 62,209) (a)


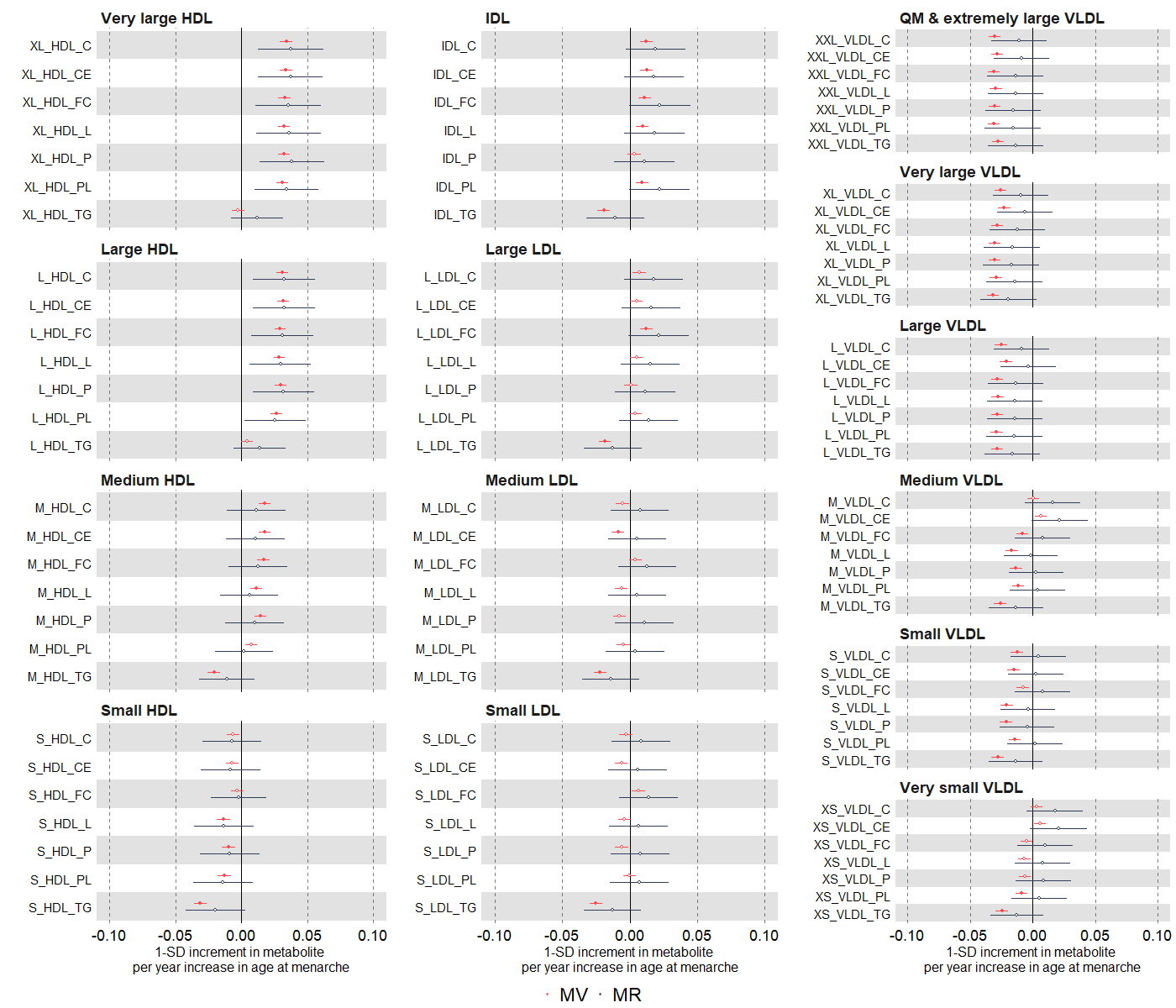


# Suppl Fig 1. (b)


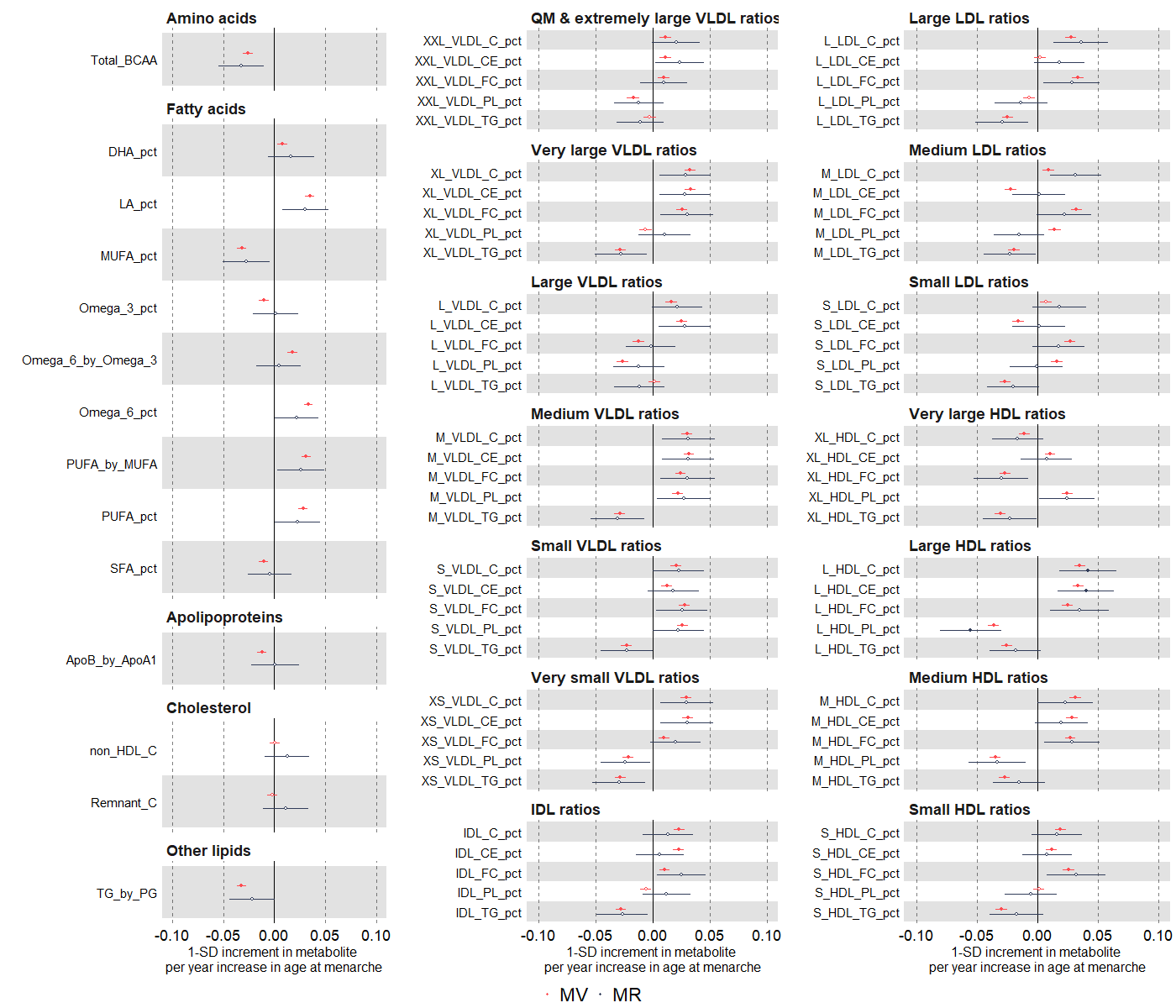


# Suppl Fig 2. Multivariable regression estimates for the relation between older age at menarche and metabolic measures among females (comparing different model adjustments). Footnote: Results are mean differences presented as standard unit changes in metabolic measure per 1 year increase in age at menarche. Circles denote the mean differences and indicate p-value < 0.00093 (filled circles) or ≥ 0.00093 (hollow circles). Horizontal bars denote 95% confidence intervals. Multivariable regression models (ordinary least squares, two-sided regression coefficients reported) were used: model 1 (unadjusted) (red, N= 63,332); model 2 (main model, age at baseline, education, and body composition at age 10) (green, N=61,920)); model 3 (additionally adjusted for BMI, smoking and alcohol status at baseline) (black, N=61,505) (a)


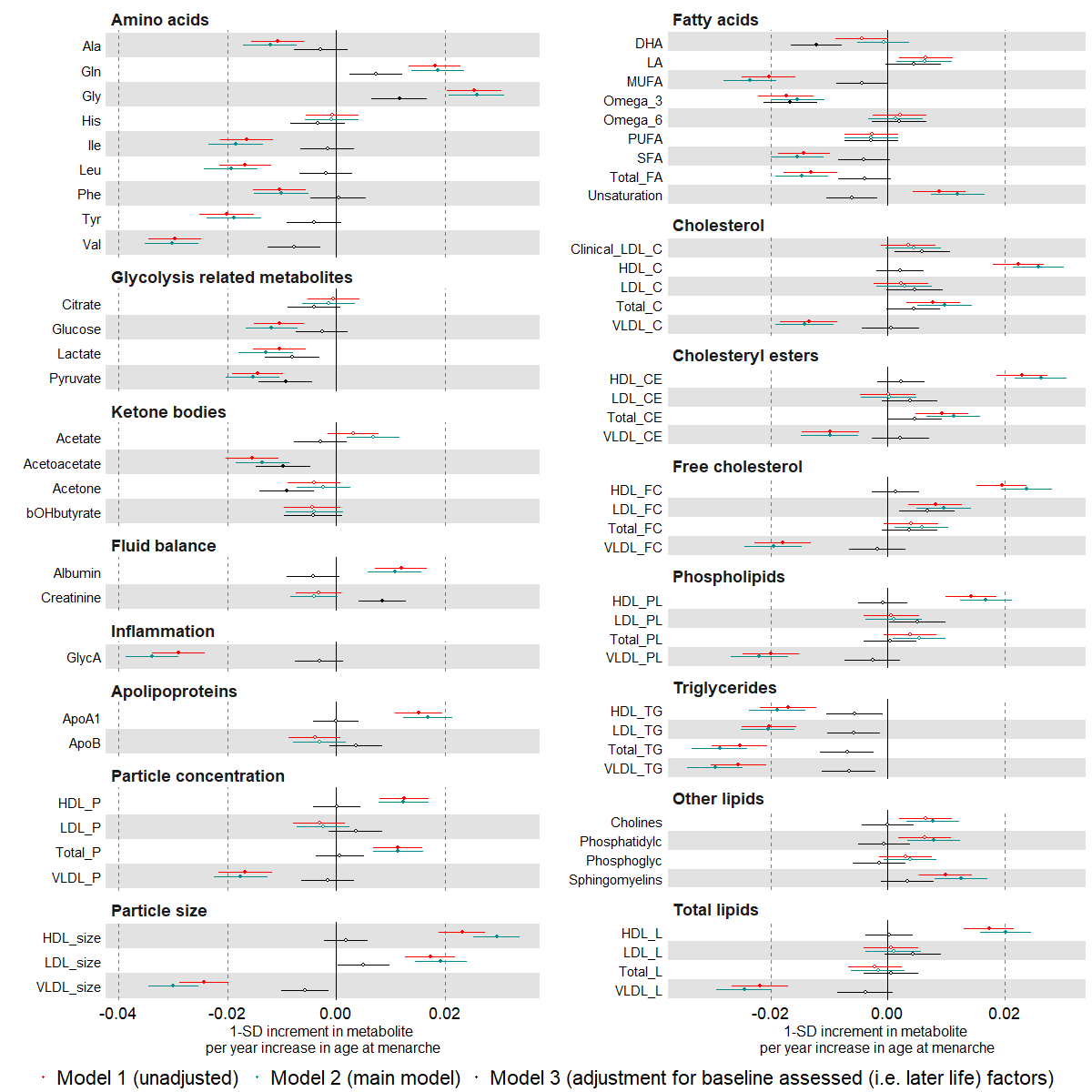


# Suppl Fig 2. (b)


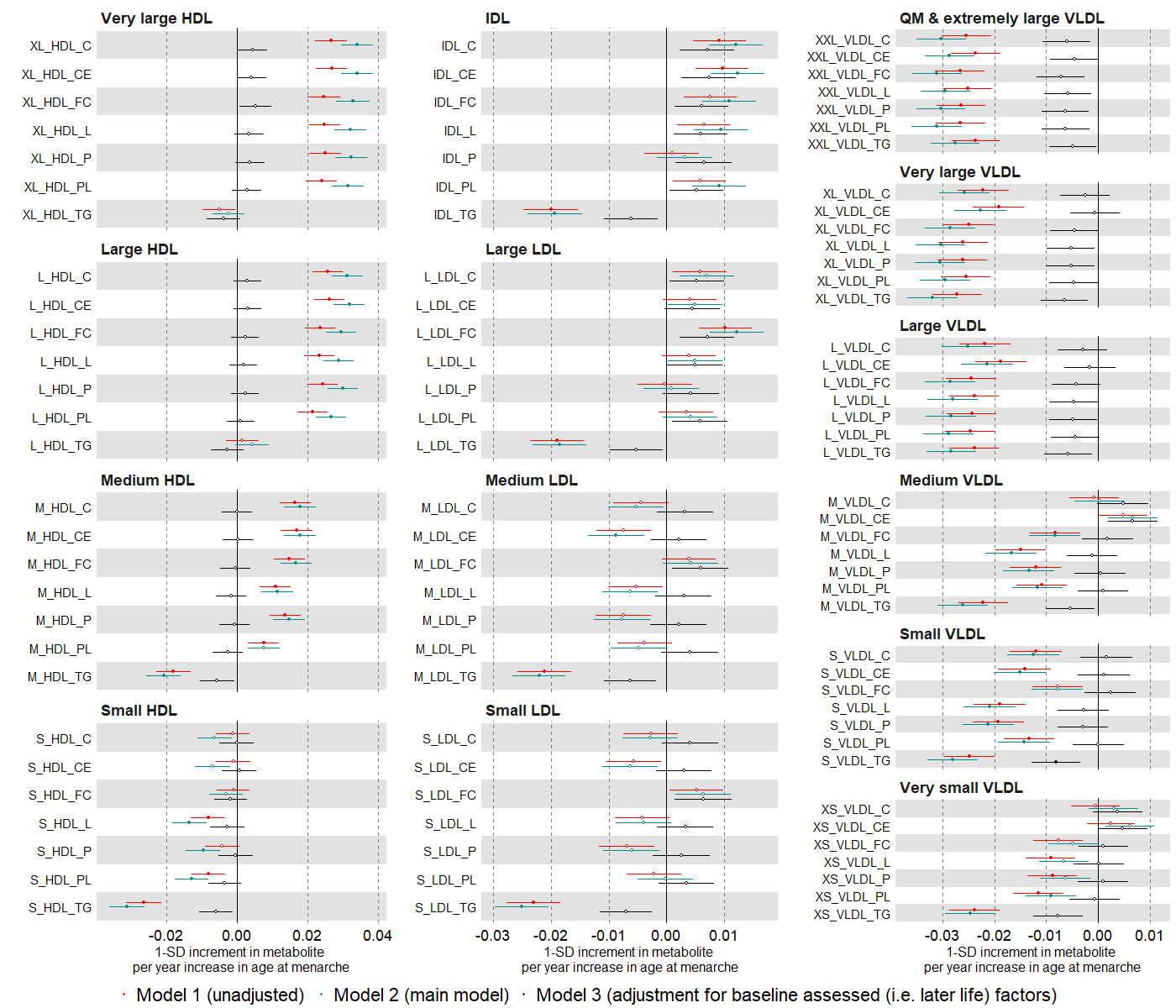


# Suppl Fig 2. (c)


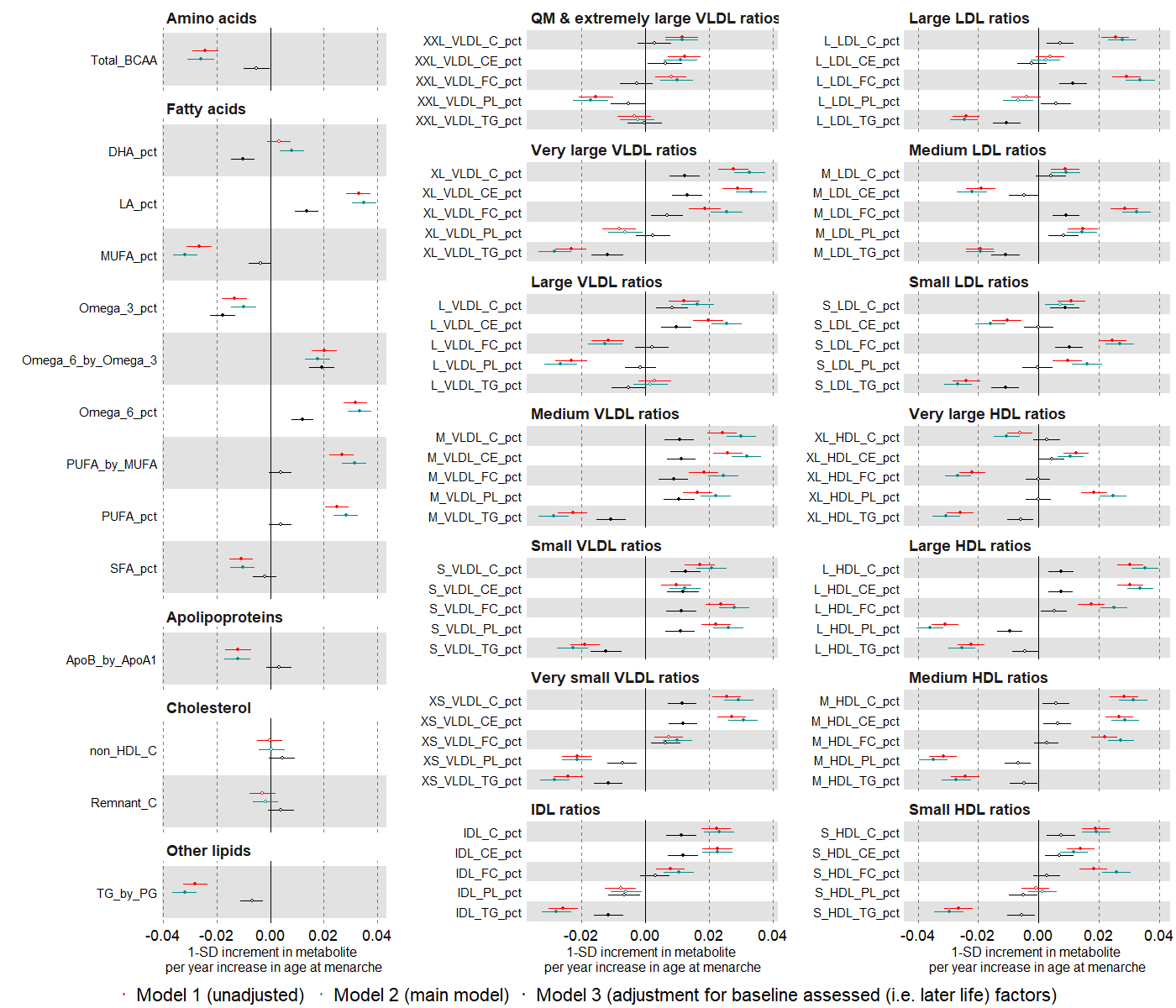


# Suppl Fig 3. Multivariable regression estimates for the relation between age at menarche (categorised: <13 years (reference), 13-14 years, >14 years) and metabolic measures among females. Footnote: Results are mean differences presented as standard unit changes in metabolic measure for age at menarche 13-14 years vs <13 years and >14 years vs <13 years, respectively. Circles denote the mean differences and indicate p-value < 0.00093 (filled circles) or ≥ 0.00093 (hollow circles). Horizontal bars denote 95% confidence intervals. Multivariable regression models (ordinary least squares, two-sided regression coefficients reported) were adjusted for age at recruitment, body size at age 10 and education (N=61,920) (a)


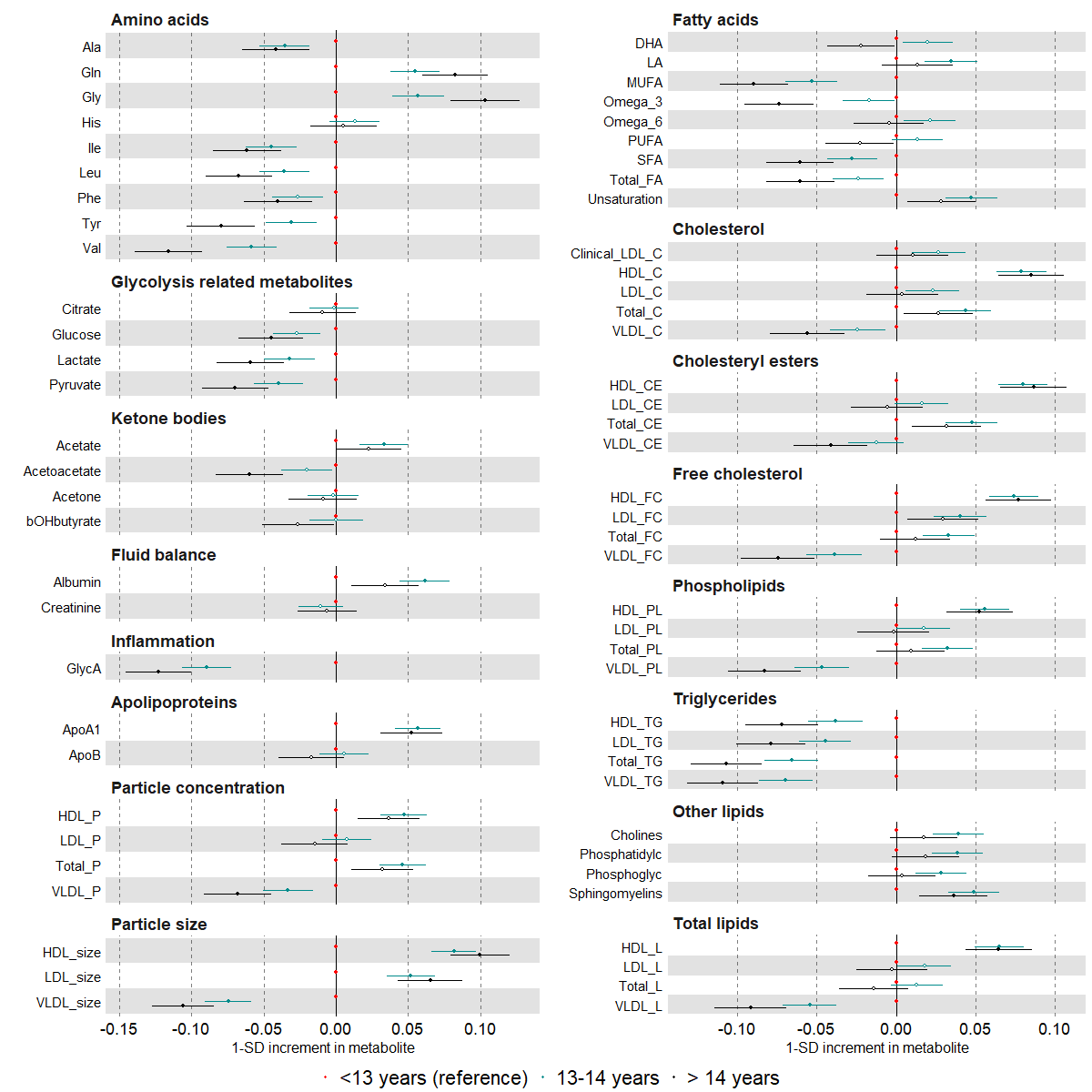


# Suppl Fig 3. (b)


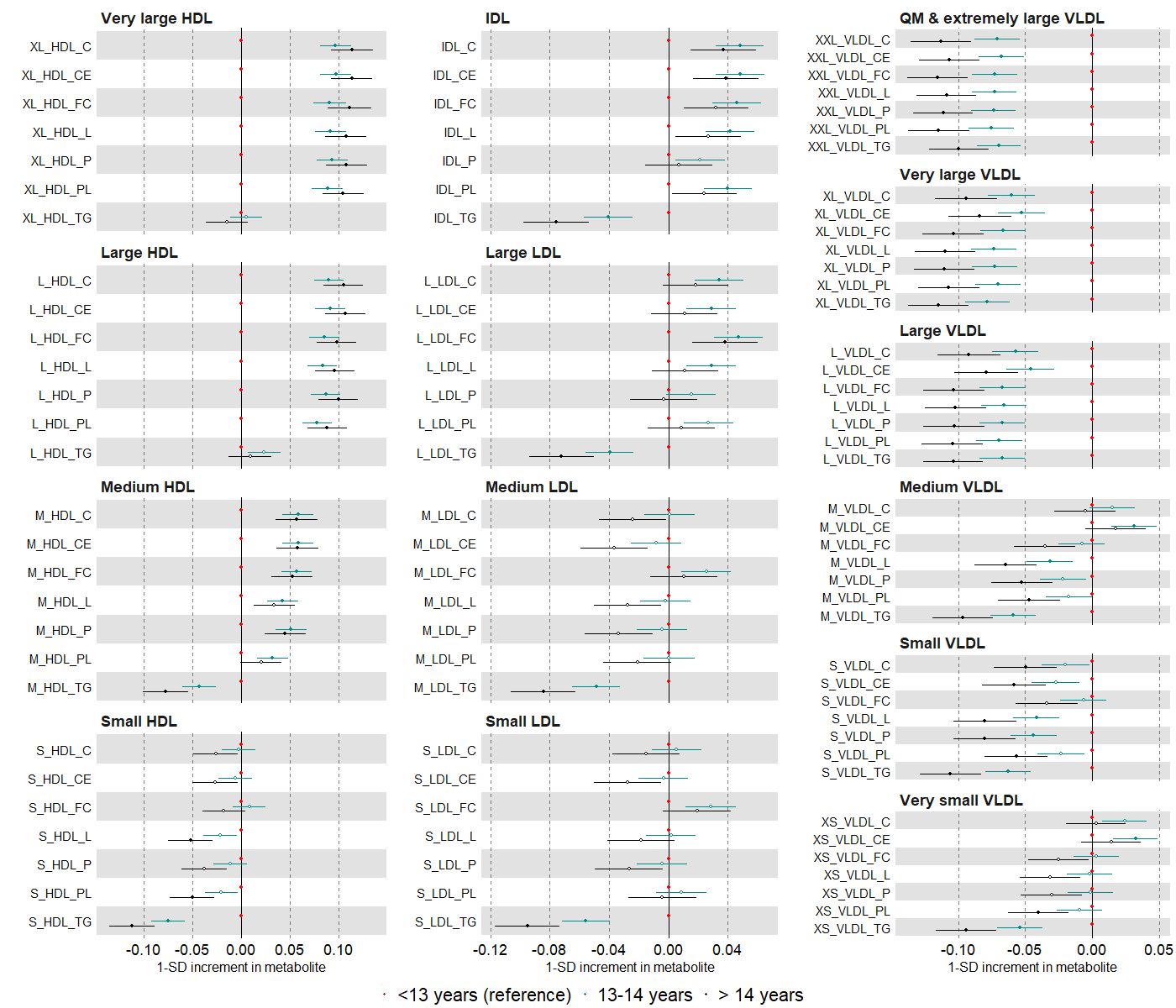


# Suppl Fig 3. (c)


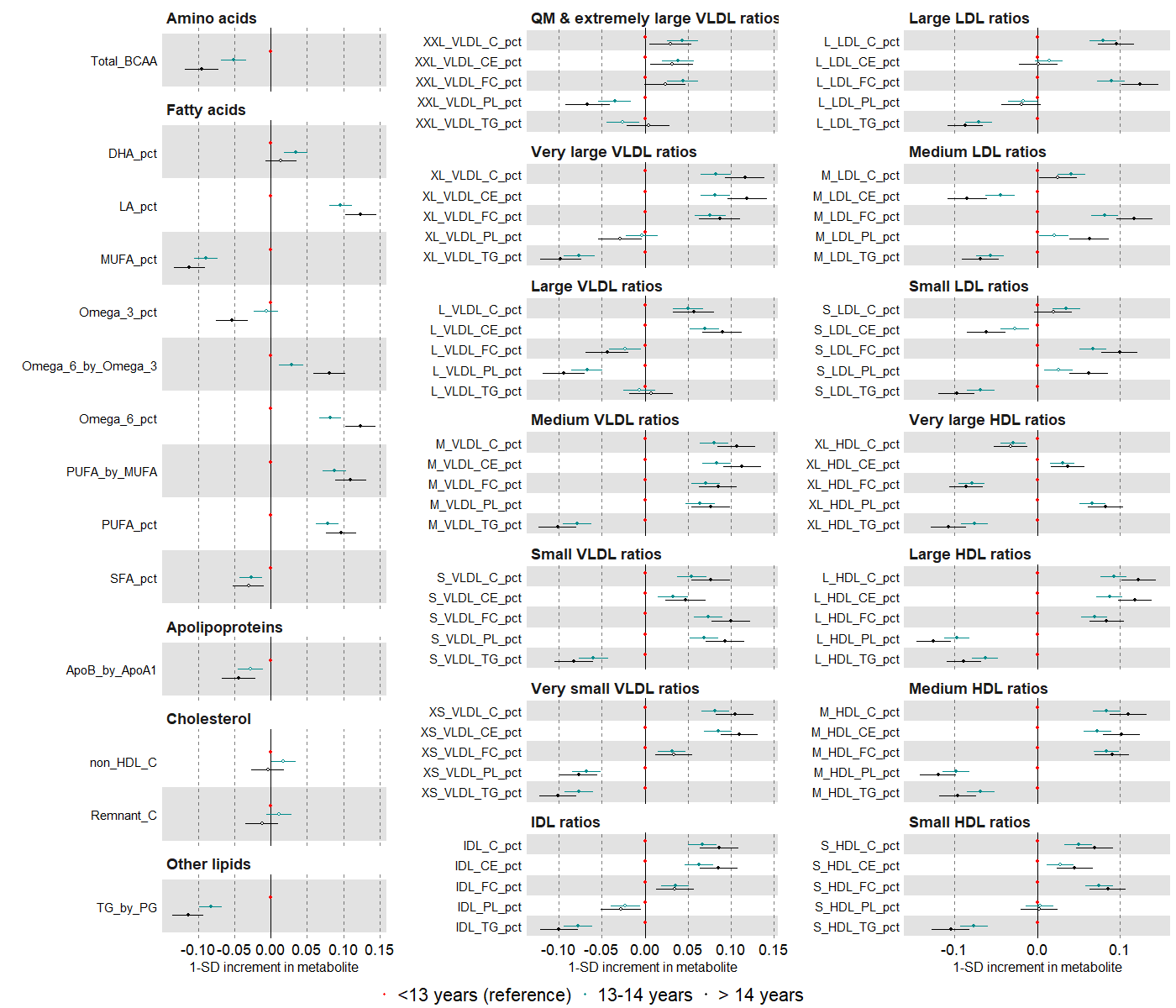


# Suppl Fig 4. Multivariable regression estimates for the relation between age at menarche (restricted cubic splines with knots placed at ages 11, 13, and 15) and metabolic measures among females. Footnote: Mean predicted outcome levels at different menarche ages for a women who is 60 years old, had an average body size at age 10 and is educated to college or university level, restricted cubic splines plotted in blue and a linear association from our main model in red (N=61,920). Error bars denote 95% confidence intervals (a)


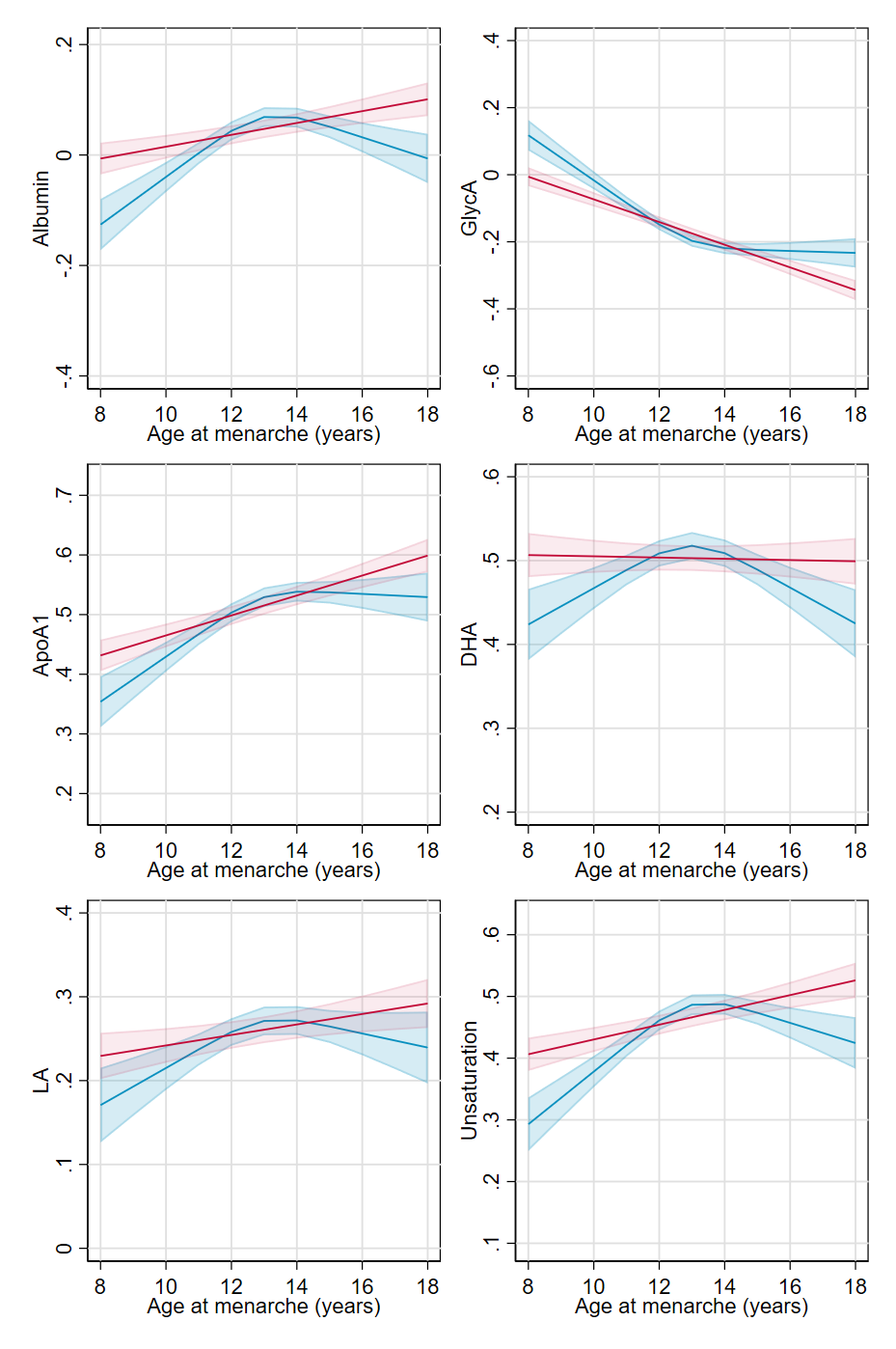


# Suppl Fig 4. (b)


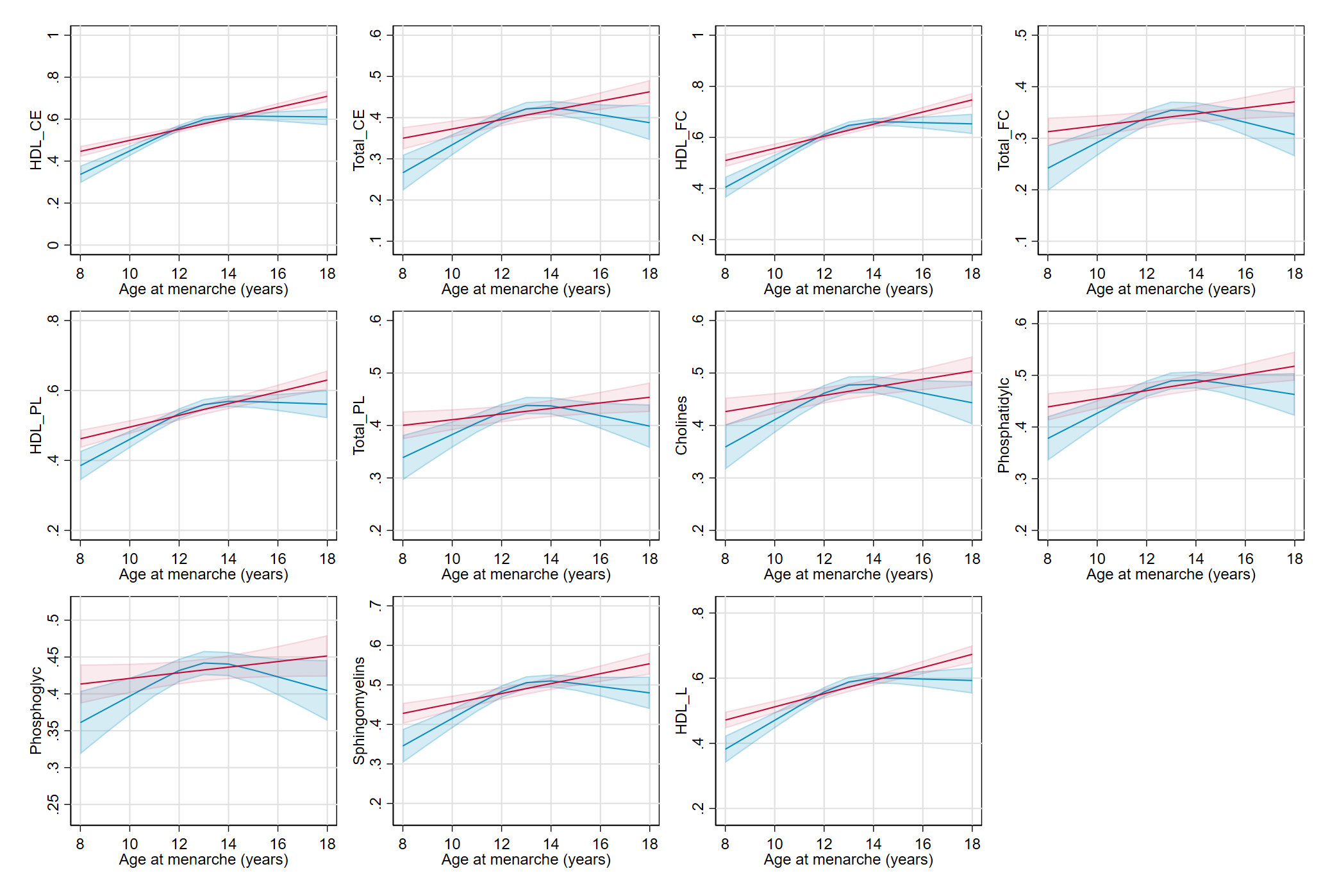


# Suppl Fig 5. Univariable and multivariable Mendelian randomization estimates for the relation between older age at menarche and NMR metabolomics measures among females. Footnote: Mendelian randomization models were estimated using the inverse variance weighted method (N= 62,209) (a)


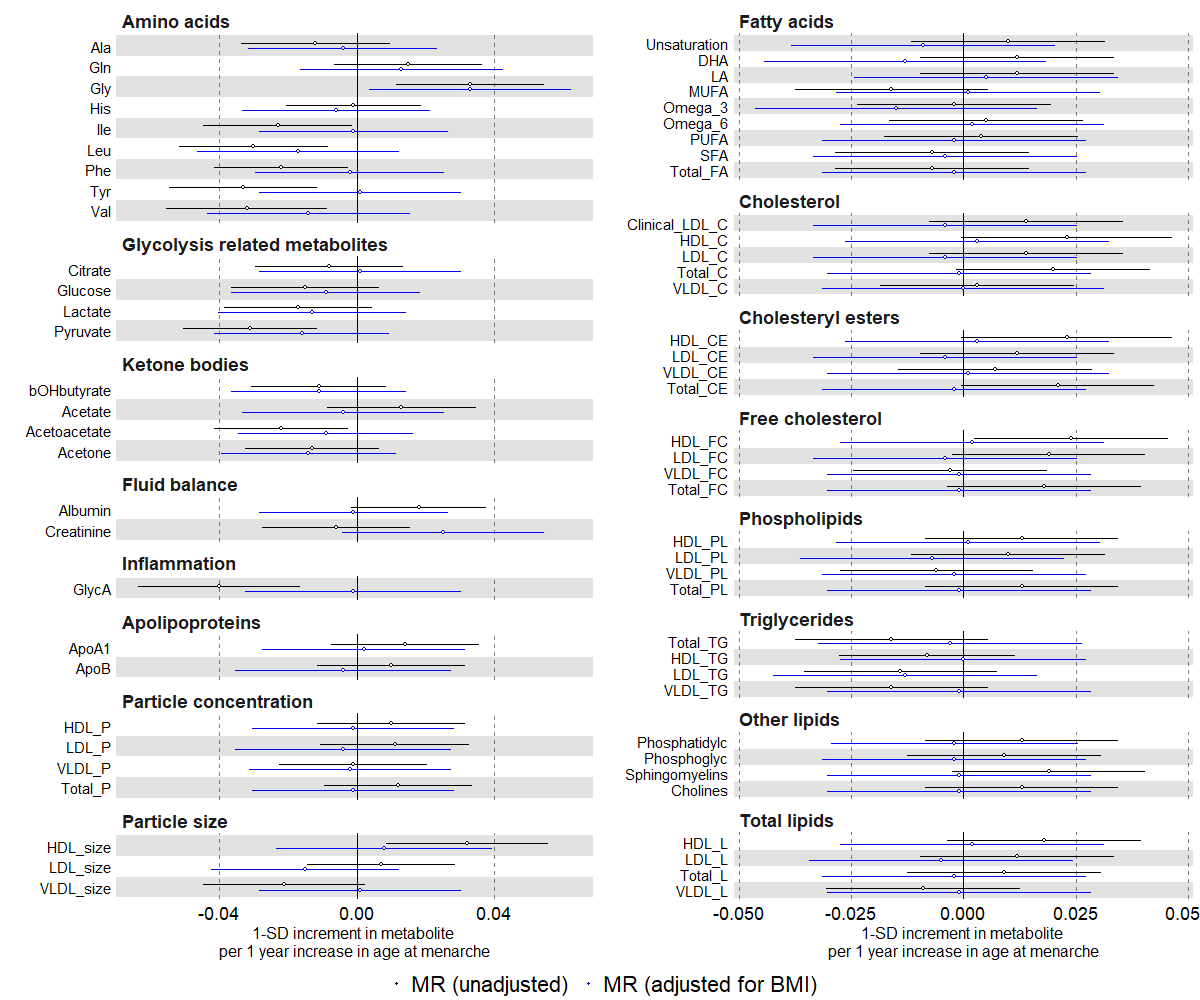


# Suppl Fig 5. (b)


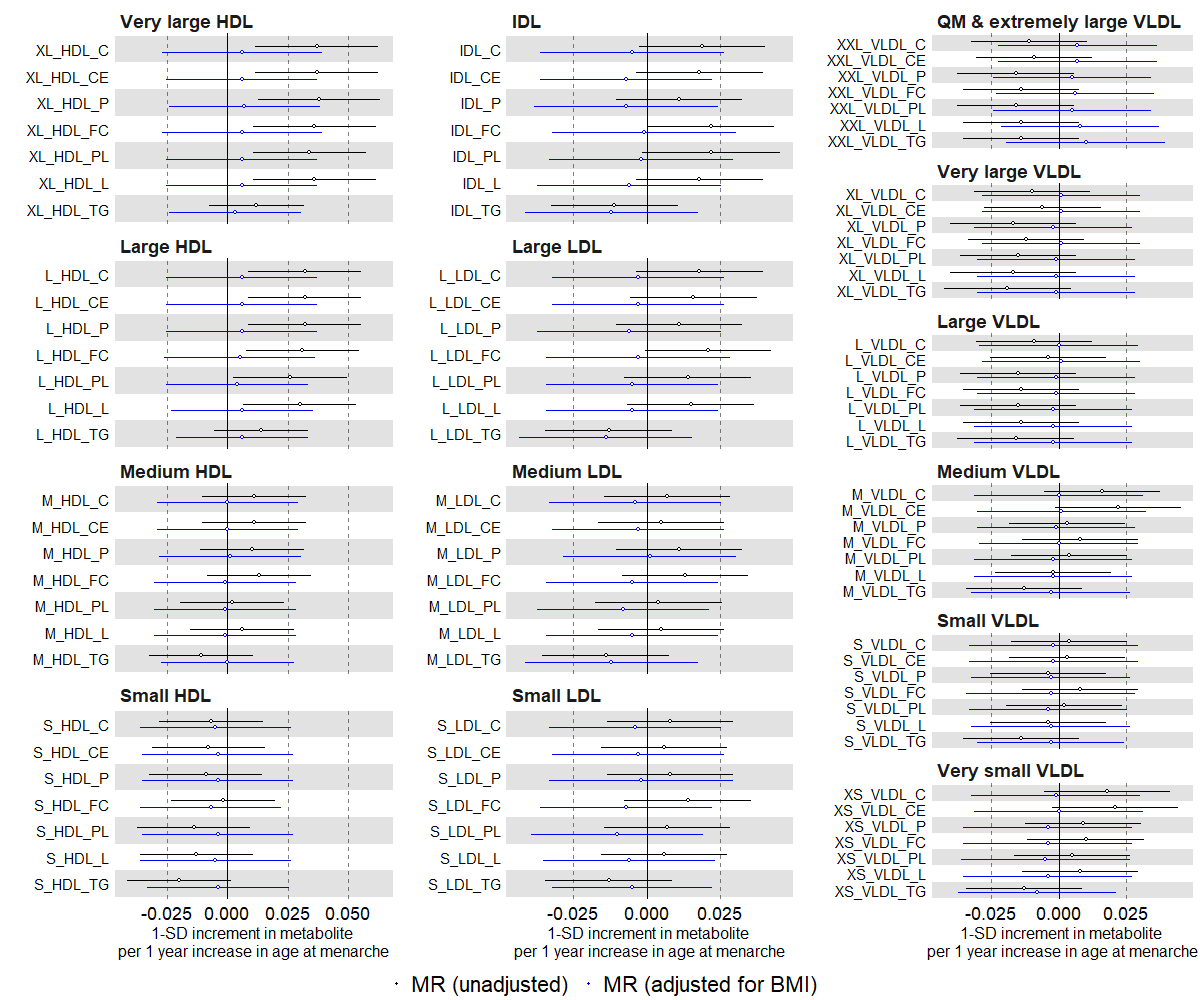


# Suppl Fig 5. (c)


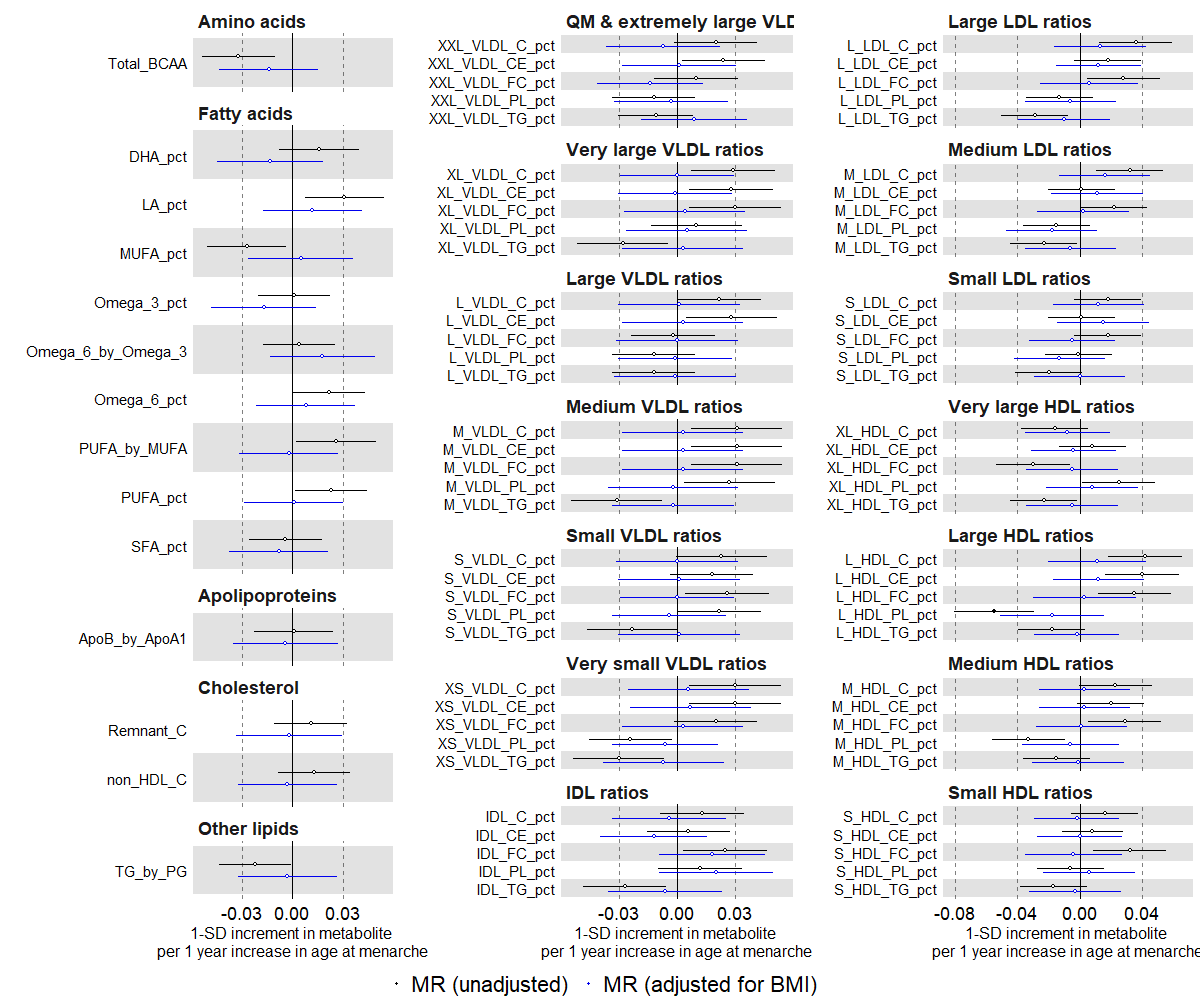


# Suppl Fig 6. Univariable and multivariable Mendelian randomization estimates for the relation between older age at menarche and clinical chemistry biomarkers among females. Mendelian randomization models were estimated using the inverse variance weighted method (N= 239,803).


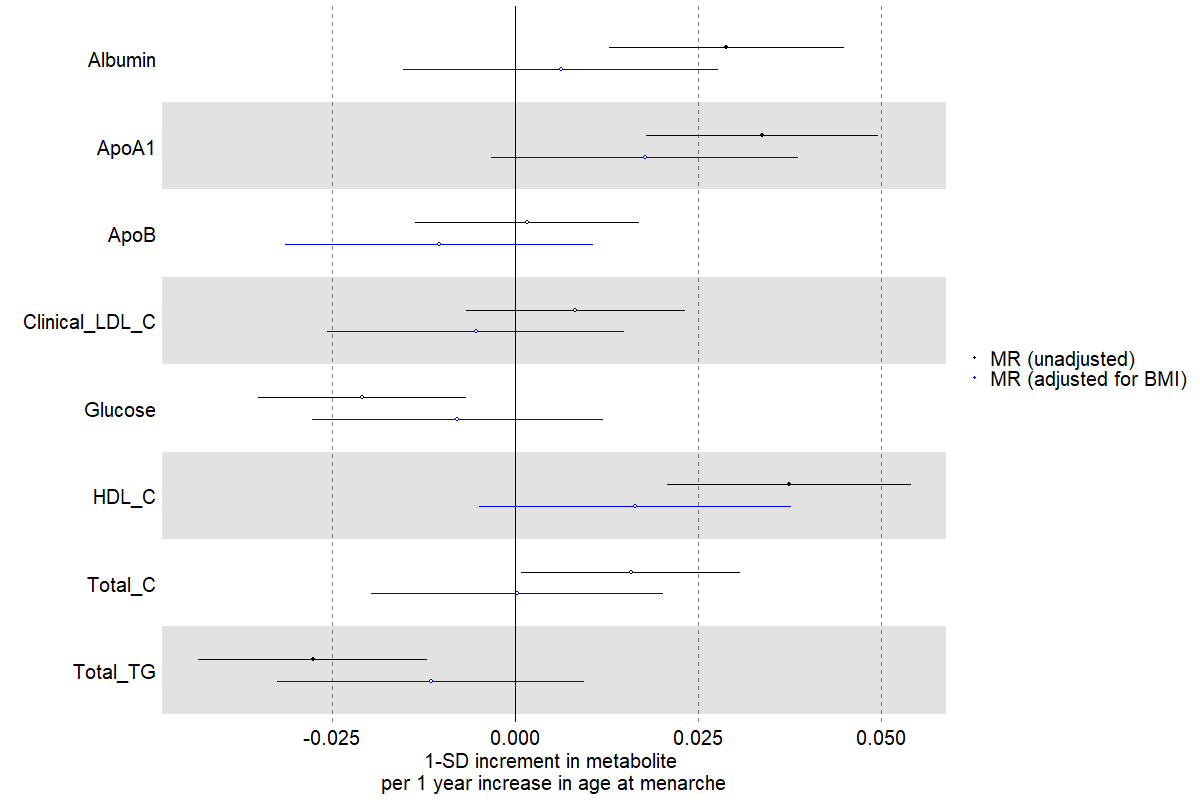


# Suppl Fig 7. Multivariable regression (red) and Mendelian randomization (black) estimates for the relation between higher parity and metabolic measures among females. Footnote: MV=multivariable; MR=Mendelian Randomisation. Results are mean differences presented as standard unit changes in metabolic measure per 1 additional child. Circles denote the mean differences and indicate p-value < 0.00093 (filled circles) or ≥ 0.00093 (hollow circles). Horizontal bars denote 95% confidence intervals. Multivariable regression models (ordinary least squares, two-sided regression coefficients reported) were adjusted for age at recruitment, body size at age 10 and education (N=63,652). Mendelian randomization models were estimated using the inverse variance weighted method (N= 62,209) (a)


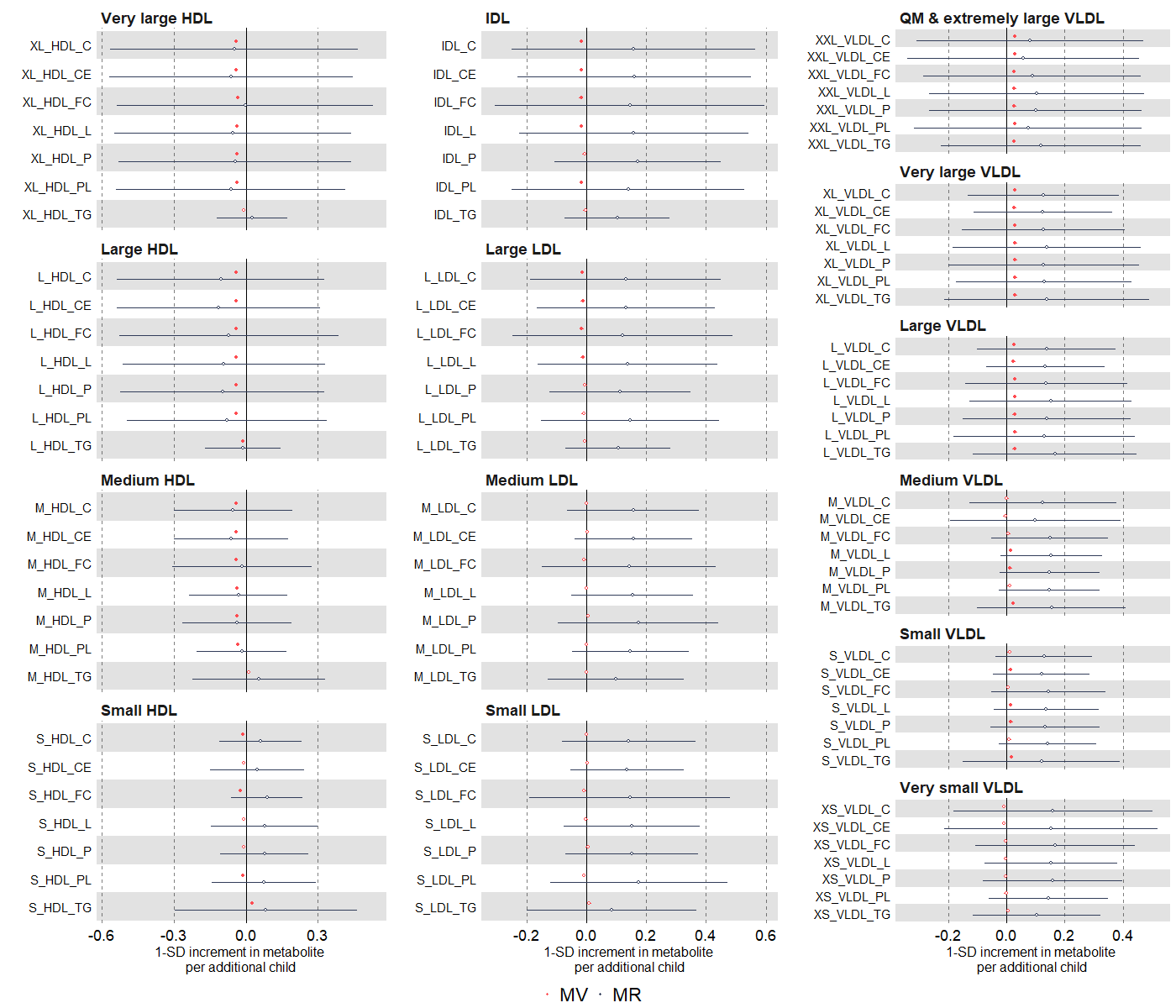


# Suppl Fig 7. (b)


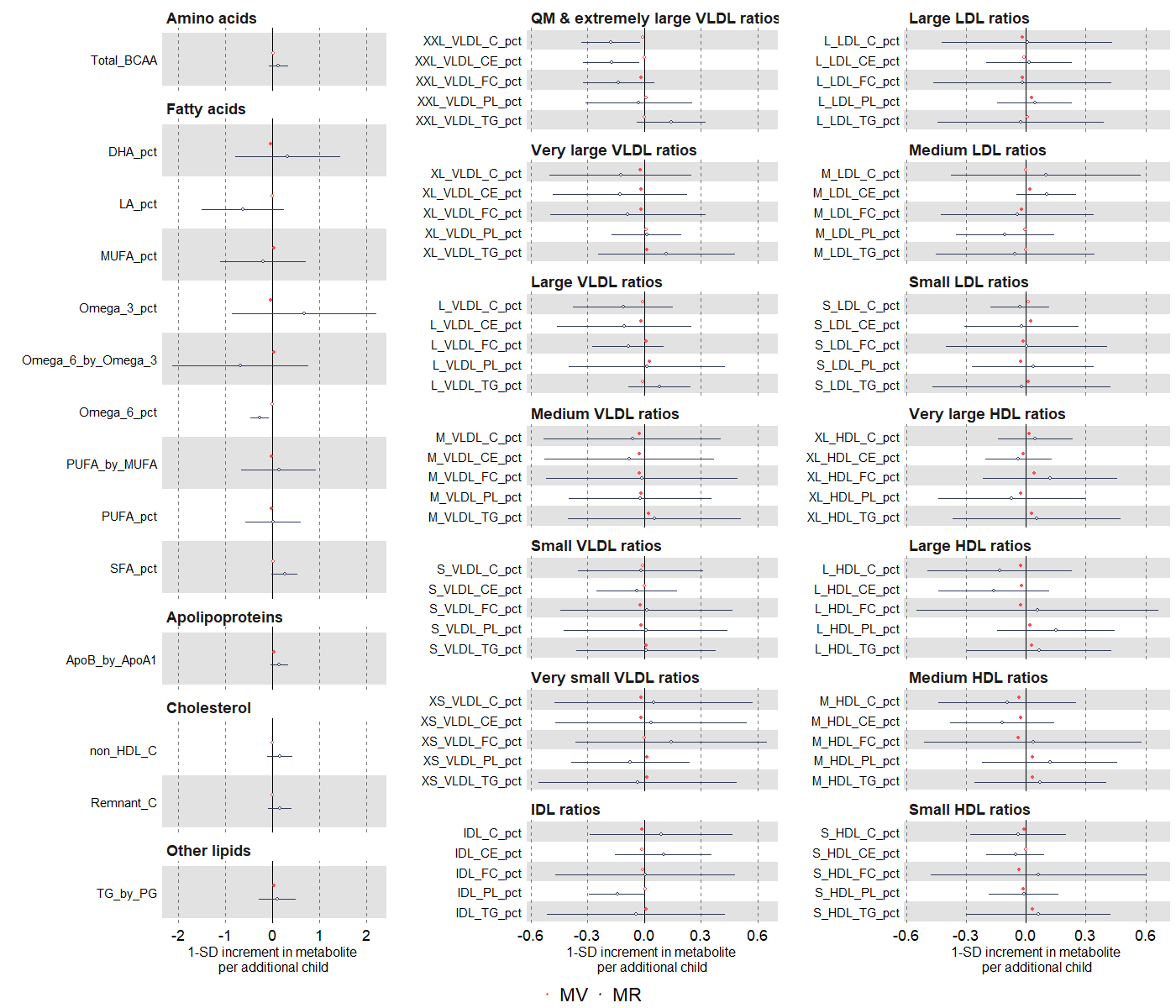


# Suppl Fig 8. Multivariable regression estimates for the relation between higher parity and metabolic measures among females (comparing different model adjustments). Footnote: Results are mean differences presented as standard unit changes in metabolic measure per additional child. Circles denote the mean differences and indicate p-value < 0.00093 (filled circles) or ≥ 0.00093 (hollow circles). Horizontal bars denote 95% confidence intervals. Multivariable regression models (ordinary least squares, two-sided regression coefficients reported) were used: model 1 (unadjusted) (red, N= 65,253); model 2 (main model, age at baseline, education, and body composition at age 10) (green, N=63,652)); model 3 (additionally adjusted for BMI, smoking and alcohol status at baseline) (black, N=63,215) (a)


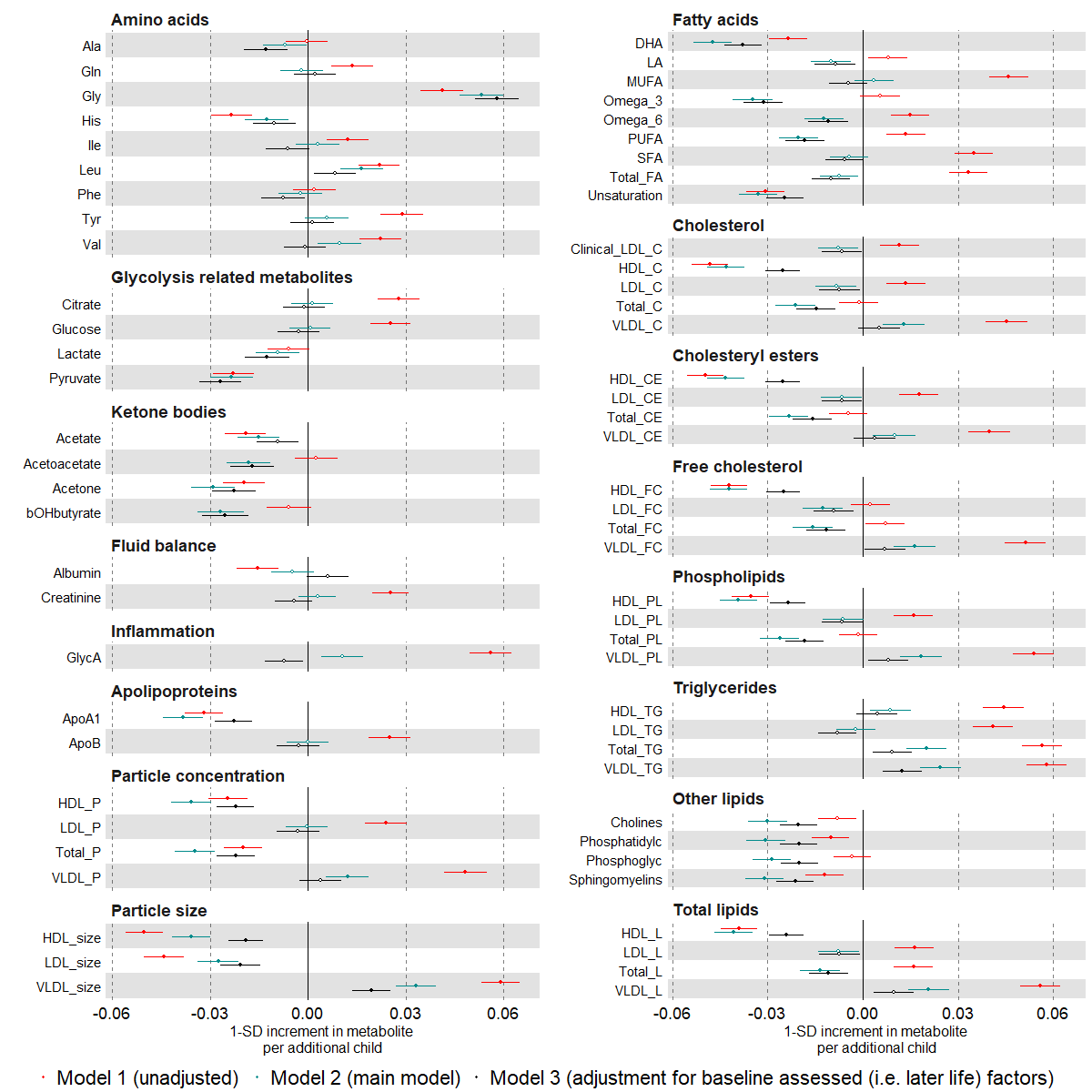


# Suppl Fig 8. (b)


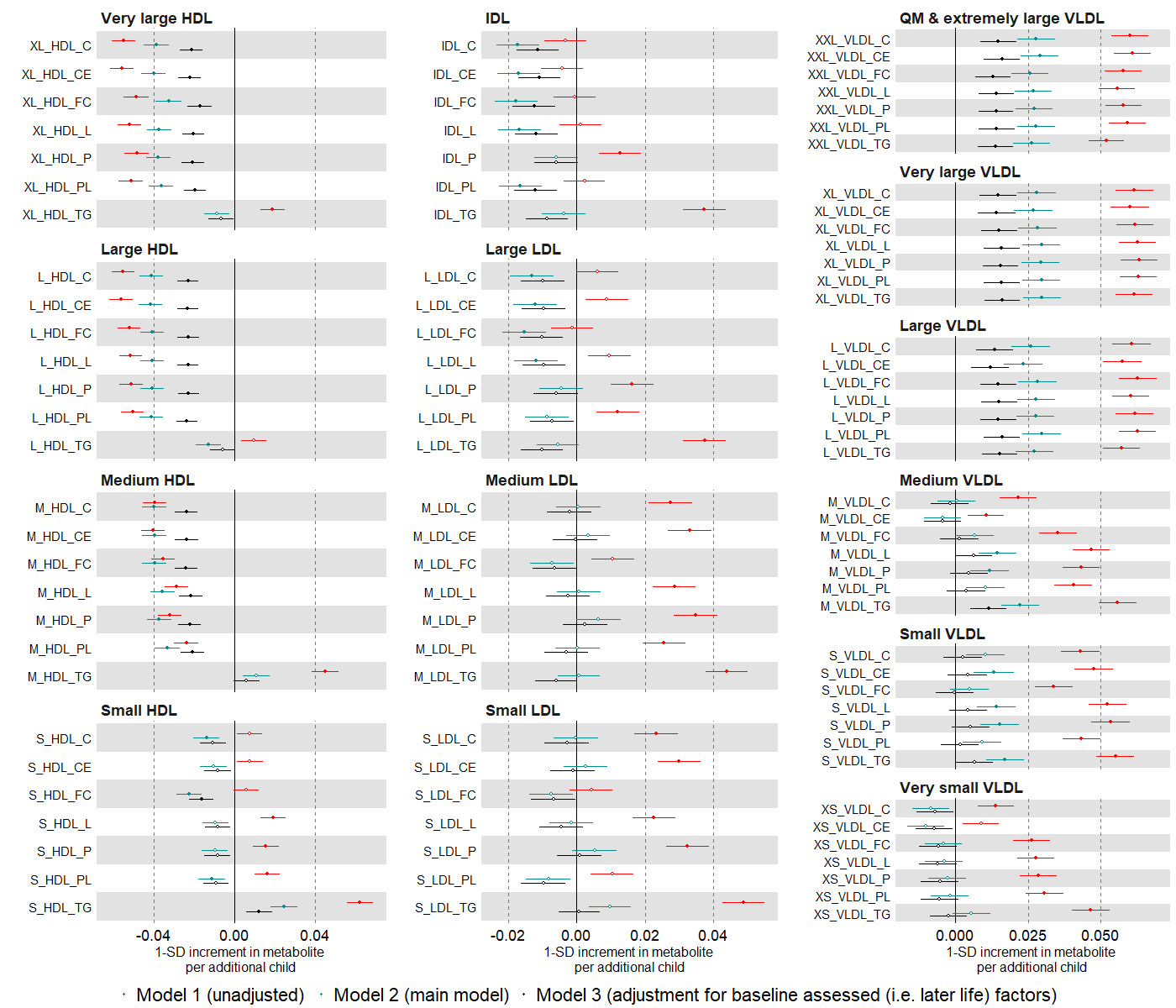


# Suppl Fig 8. (c)


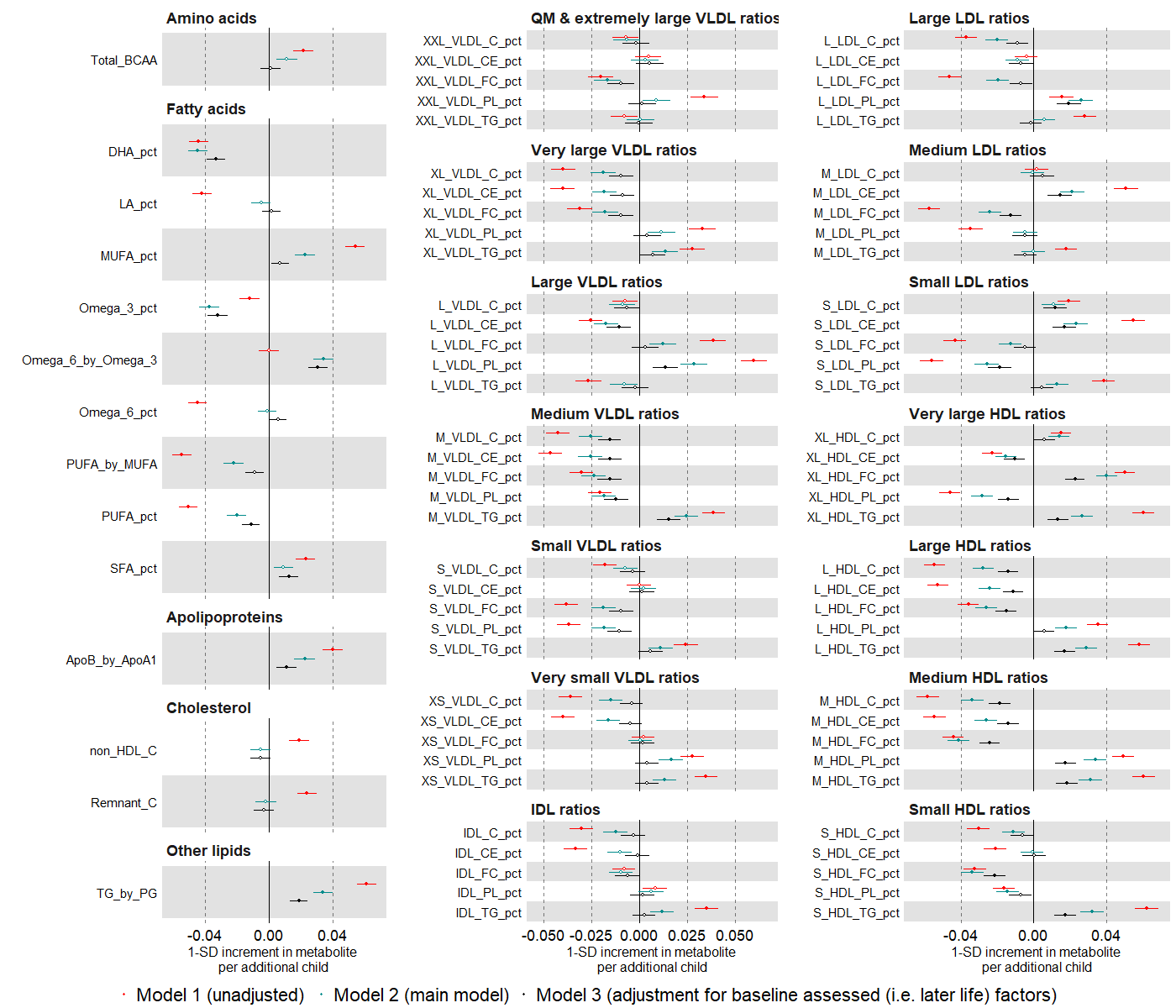


# Suppl Fig 9. Multivariable regression estimates for the relation between parity (categorised: 0 (reference), 1, 2, 3+) and metabolic measures among females. Footnote: Results are mean differences presented as standard unit changes in metabolic measure for parity 1 vs 0, 2 vs 0 and 3+ vs 0, respectively. Circles denote the mean differences and indicate p-value < 0.00093 (filled circles) or ≥ 0.00093 (hollow circles). Horizontal bars denote 95% confidence intervals. Multivariable regression models (ordinary least squares, two-sided regression coefficients reported) were adjusted for age at recruitment, body size at age 10 and education (N=63,652) (a)


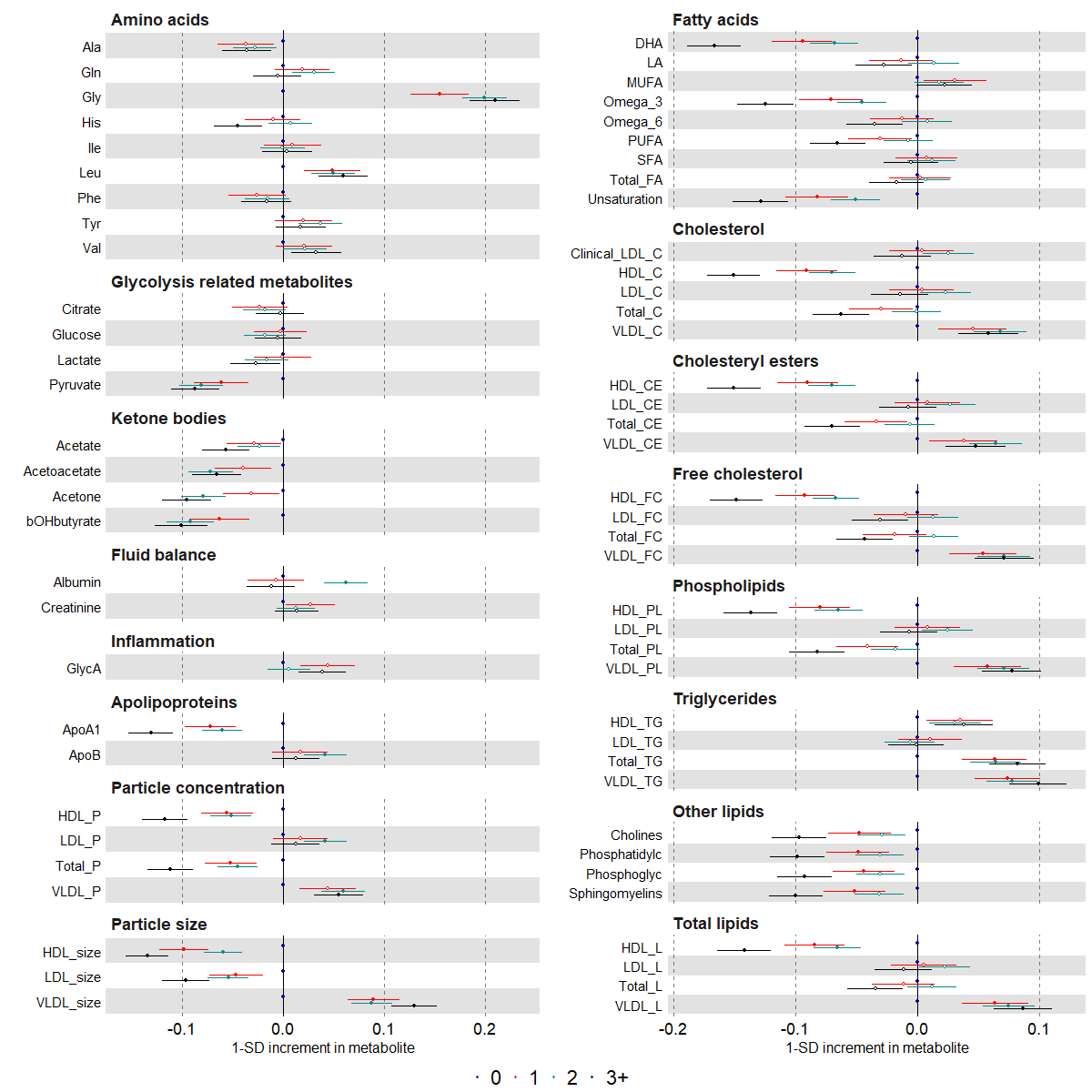


# Suppl Fig 9. (b)


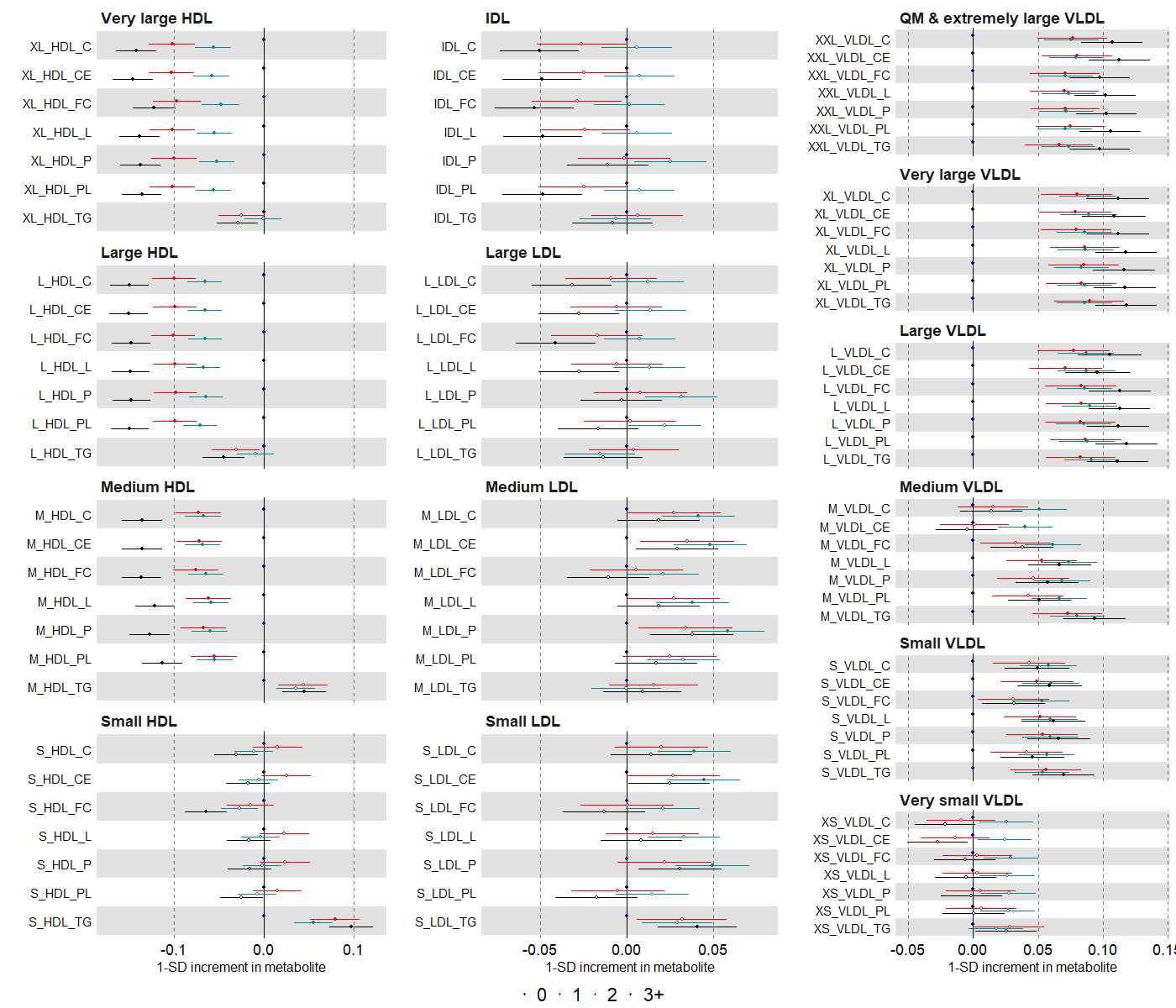


# Suppl Fig 9. (c)


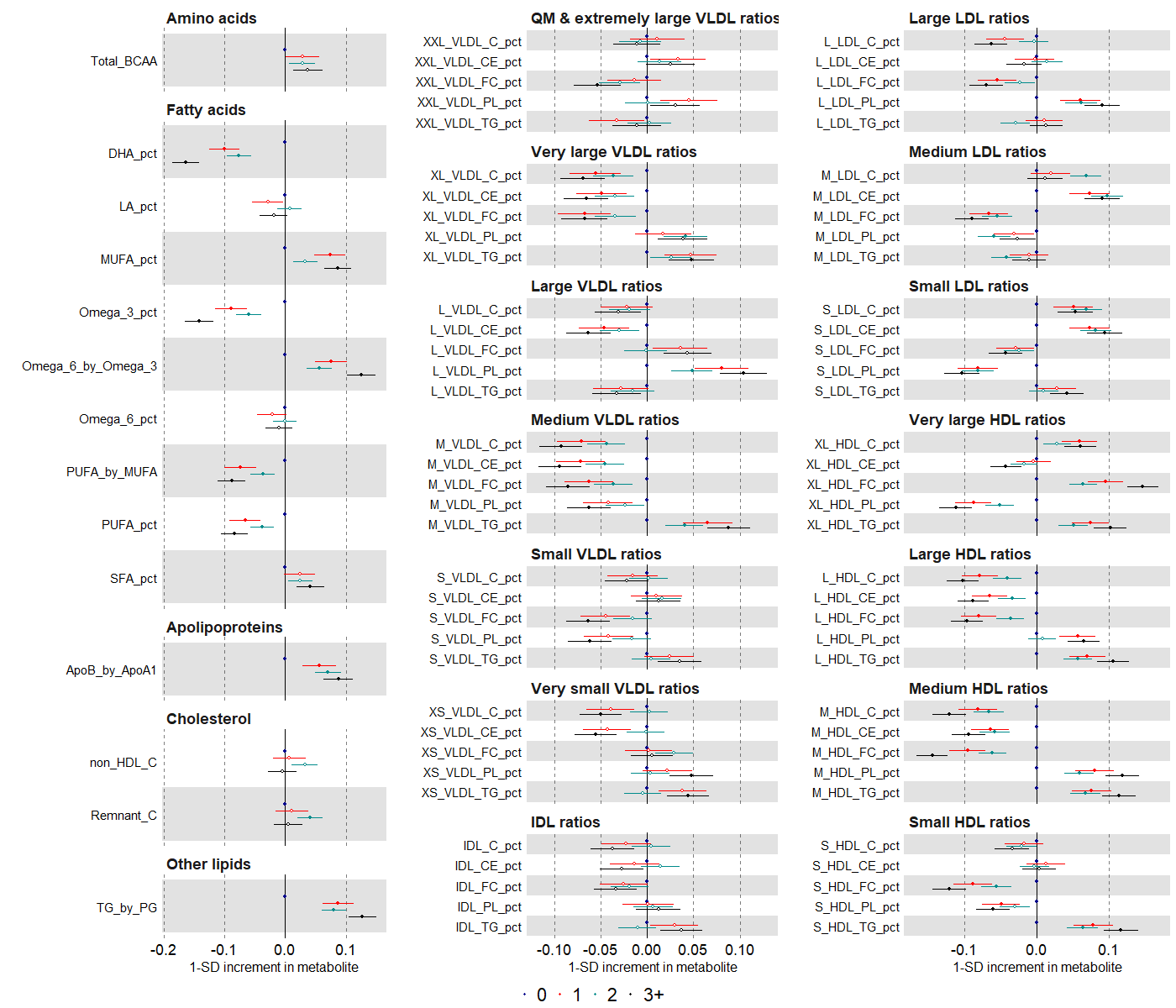


# Suppl Fig 10. Multivariable regression estimates for the relation between parity (restricted cubic splines with knots placed at 1 2 and 3) and metabolic measures among females. Footnote: Mean predicted outcome levels at different levels of parity for a women who is 60 years old, had an average body size at age 10 and is educated to college or university level (N=63,652). Error bars denote 95% confidence intervals (a)


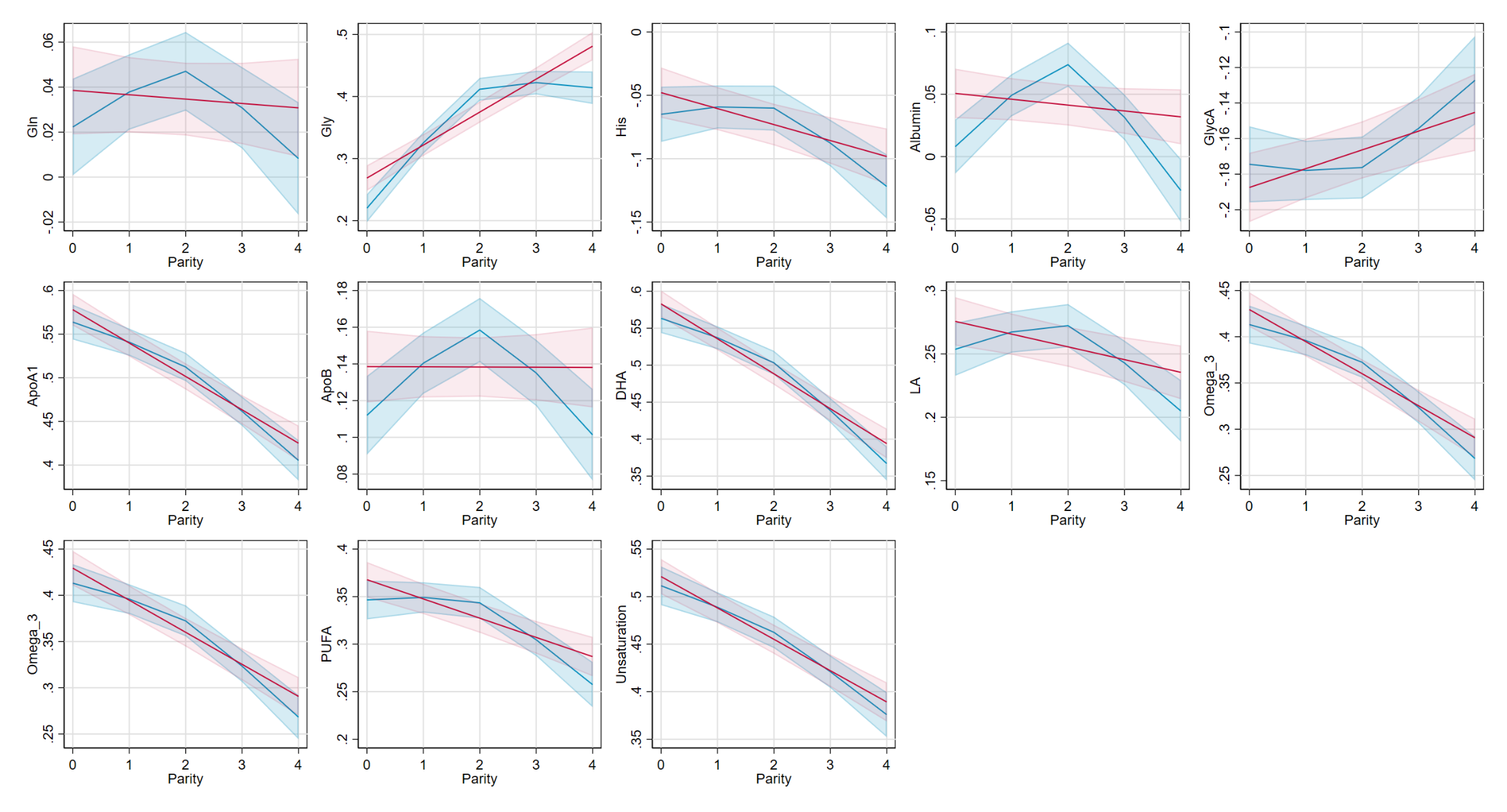


# Suppl Fig 10. (b)


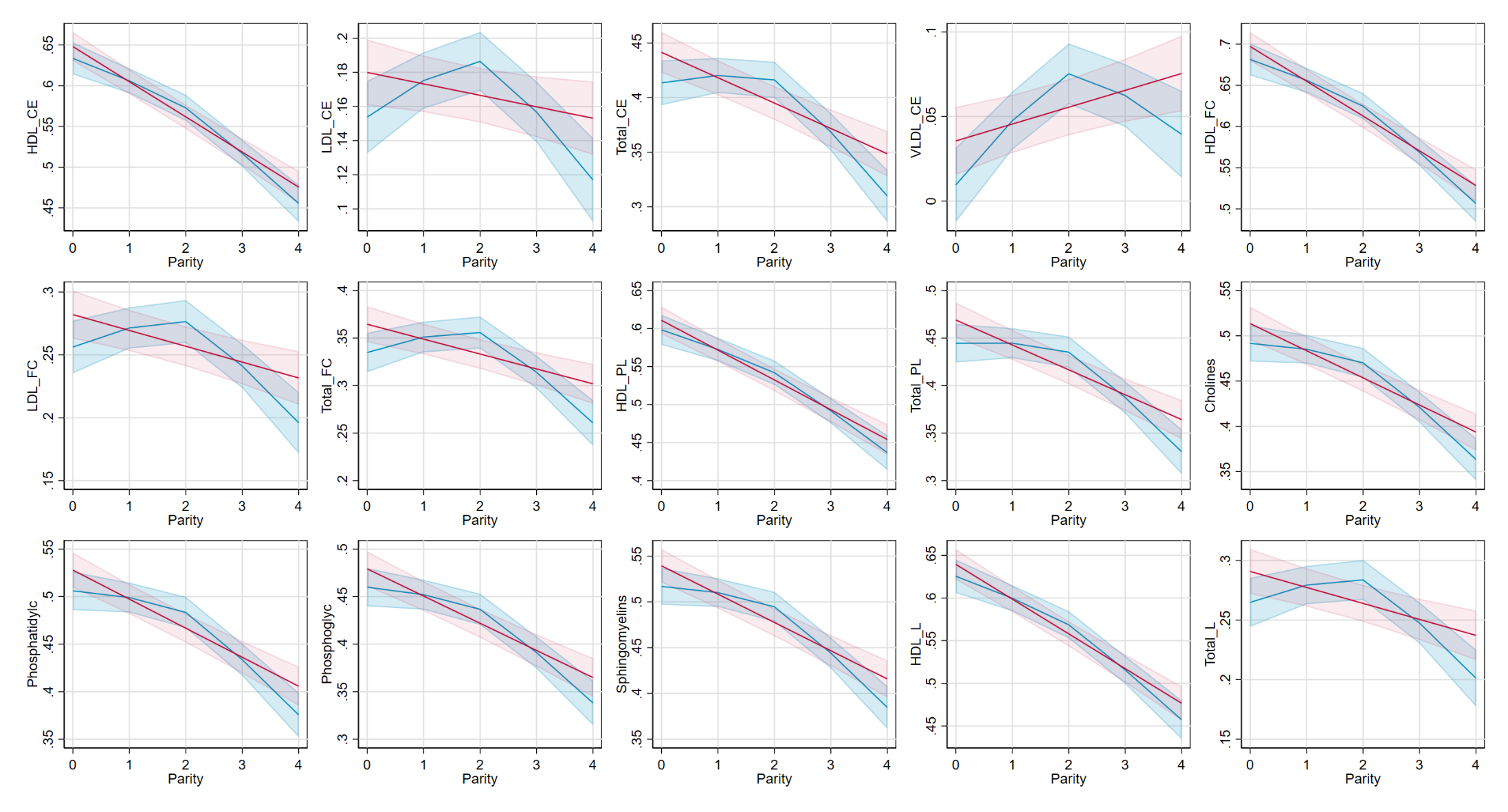


# Suppl Fig 11. Multivariable regression (red) and Mendelian randomization (black) estimates for the relation between older age at natural menopause and metabolic measures among females. Footnote: Results are mean differences presented as standard unit changes in metabolic measure per 1 year increase in age at menopause. Circles denote the mean differences and indicate p-value < 0.00093 (filled circles) or ≥ 0.00093 (hollow circles). Horizontal bars denote 95% confidence intervals. Multivariable regression models (ordinary least squares, two-sided regression coefficients reported) were adjusted for age at recruitment, body size at age 10 and education (N=36,253). Mendelian randomization models were estimated using the inverse variance weighted method (N= 62,209) (a)


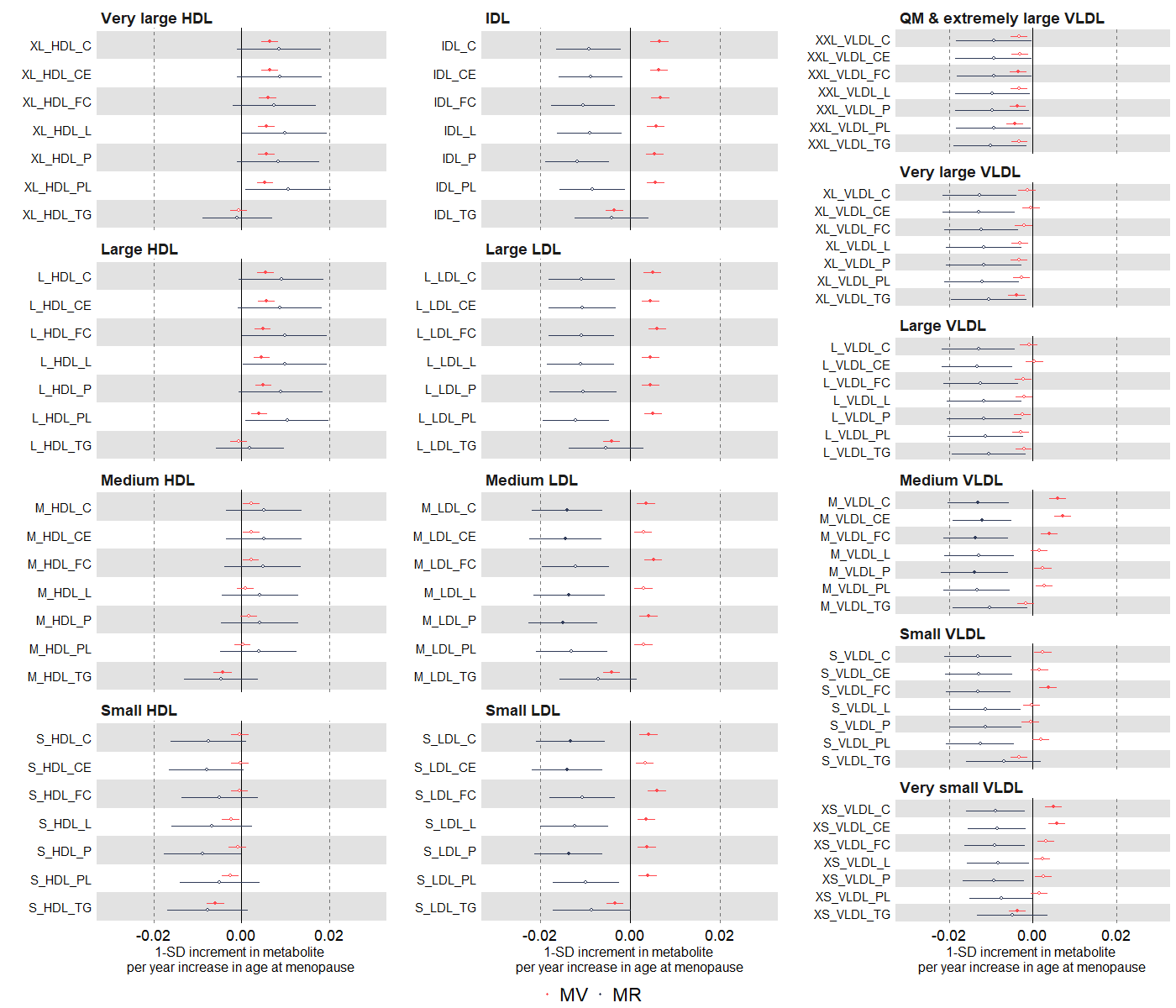


# Suppl Fig 11. (b)


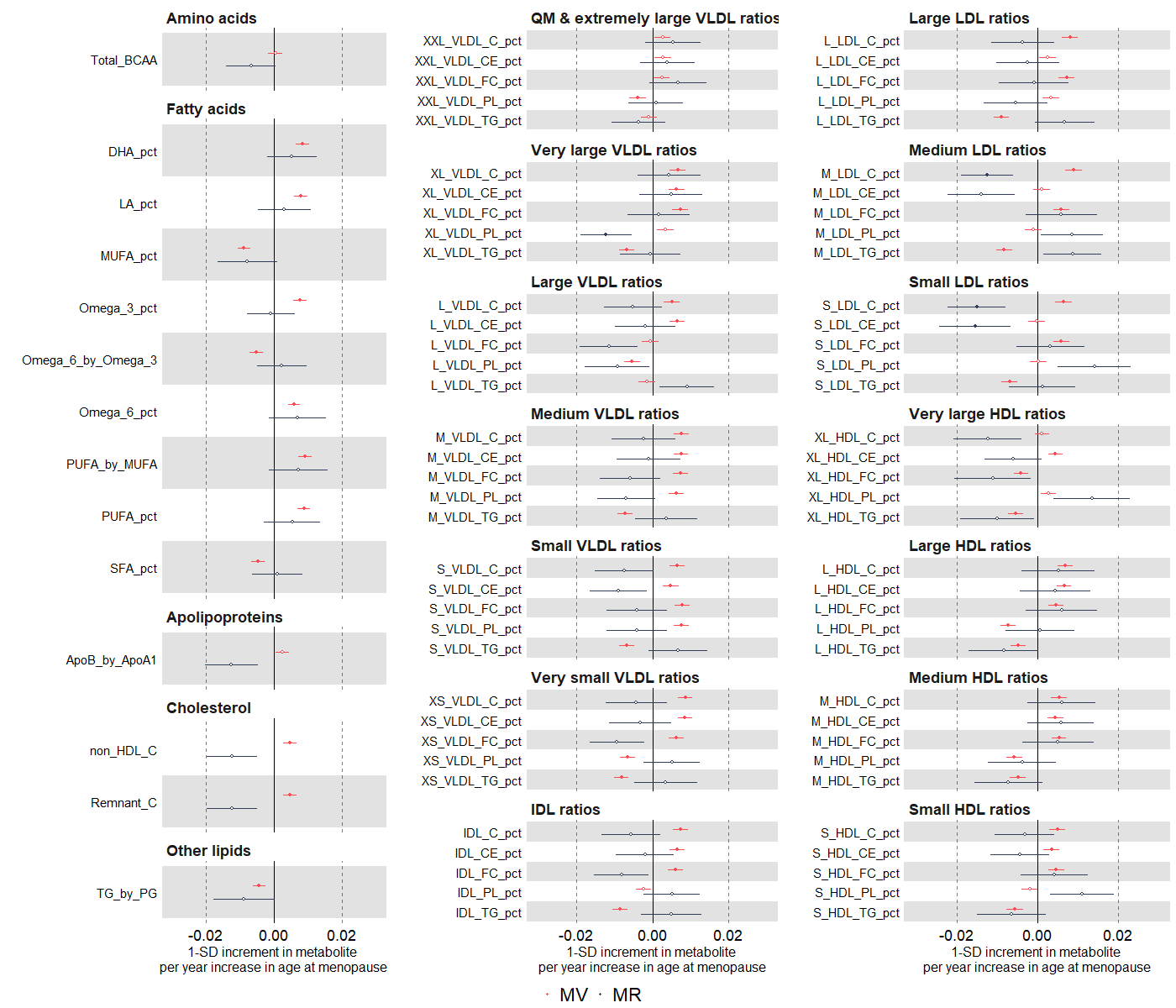


# Suppl Fig 12. Multivariable regression estimates for the relation between older age at natural menopause and metabolic measures among females (comparing different model adjustments). Footnote: Results are mean differences presented as standard unit changes in metabolic measure per 1 year increase in age at menopause. Circles denote the mean differences and indicate p-value < 0.00093 (filled circles) or ≥ 0.00093 (hollow circles). Horizontal bars denote 95% confidence intervals. Multivariable regression models (ordinary least squares, two-sided regression coefficients reported) were used: model 1 (unadjusted) (red, N= 37,112); model 2 (main model, age at baseline, education, and body composition at age 10) (green, N=36,253)); model 3 (additionally adjusted for BMI, smoking and alcohol status at baseline) (black, N=35,997) (a)


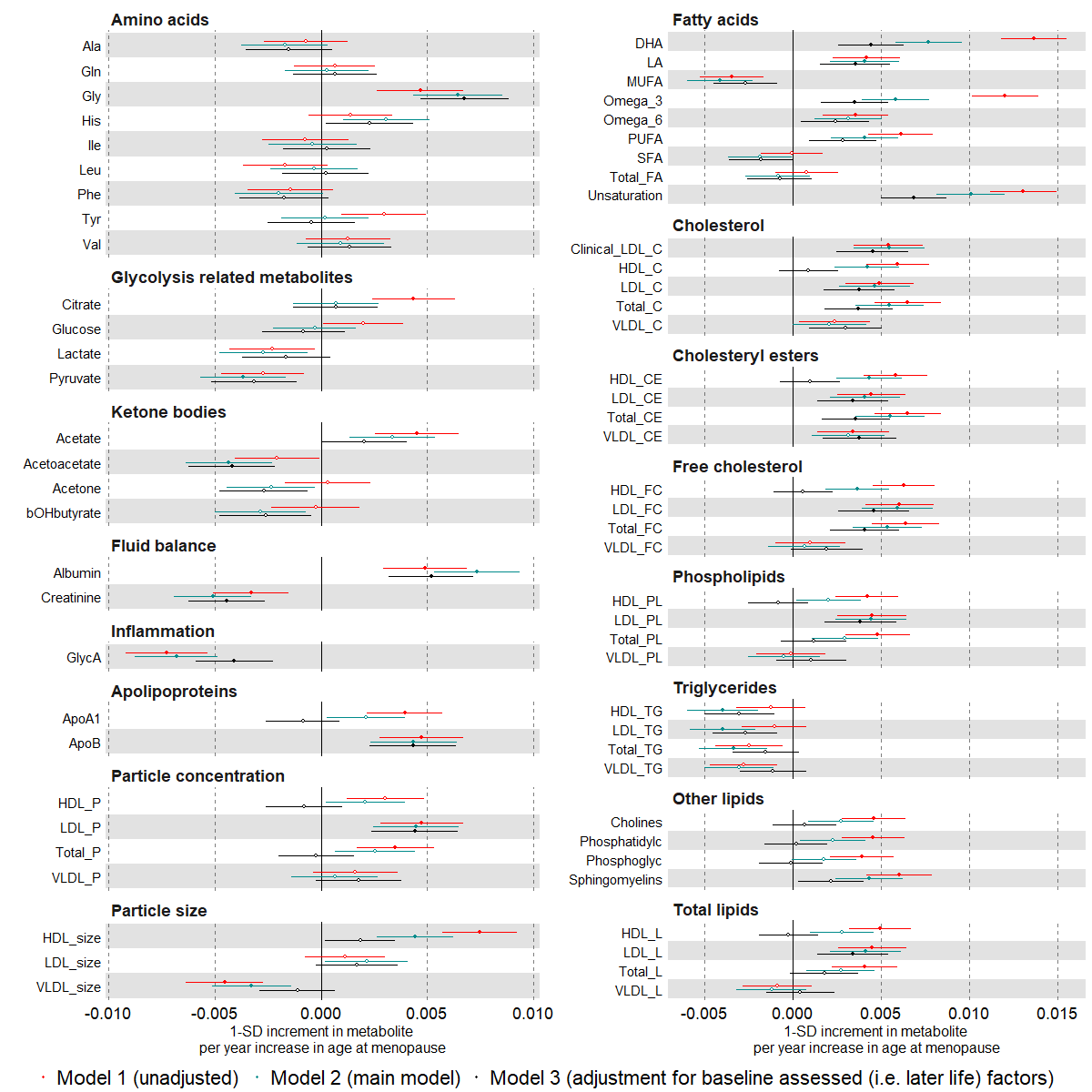


# Suppl Fig 12. (b)


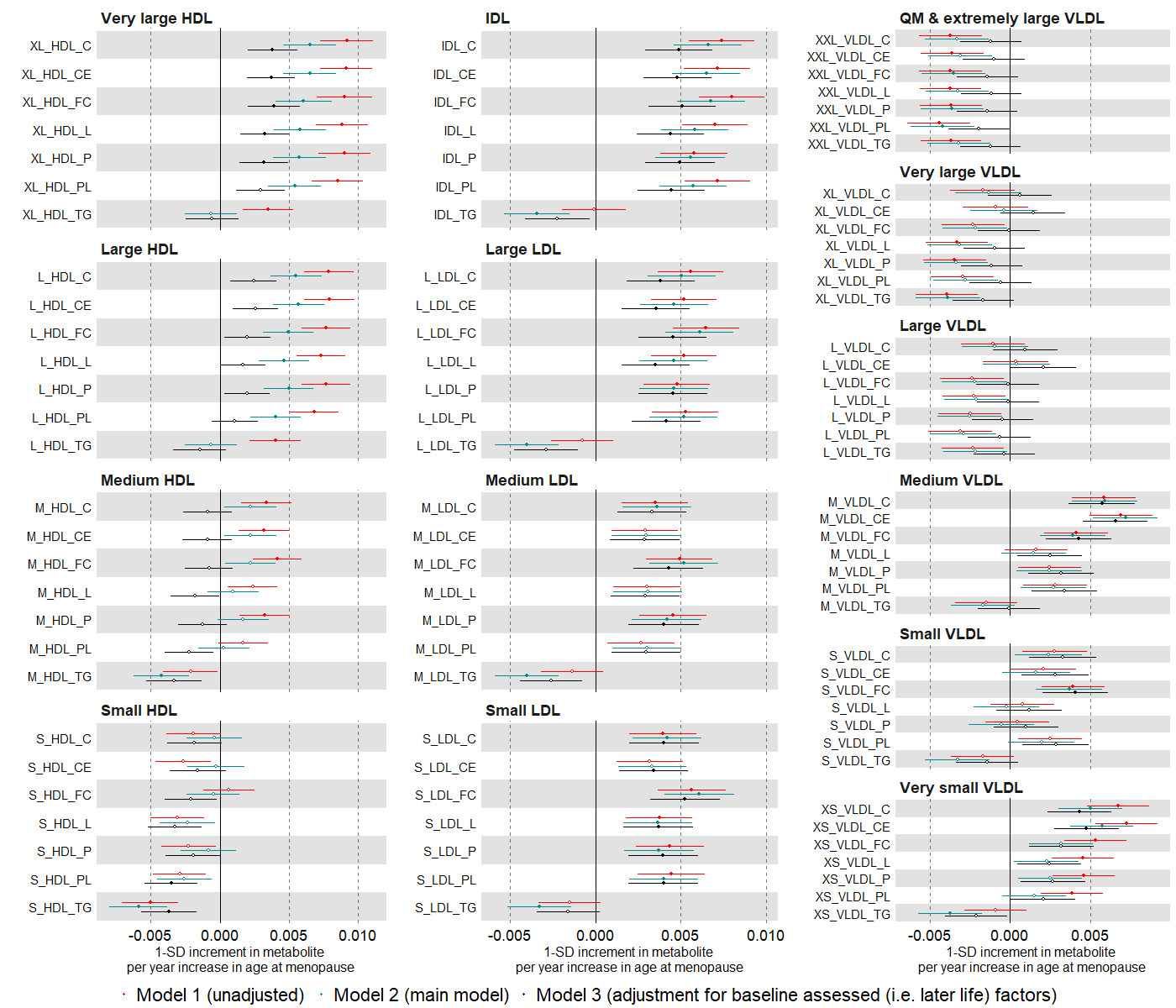


# Suppl Fig 12. (c)


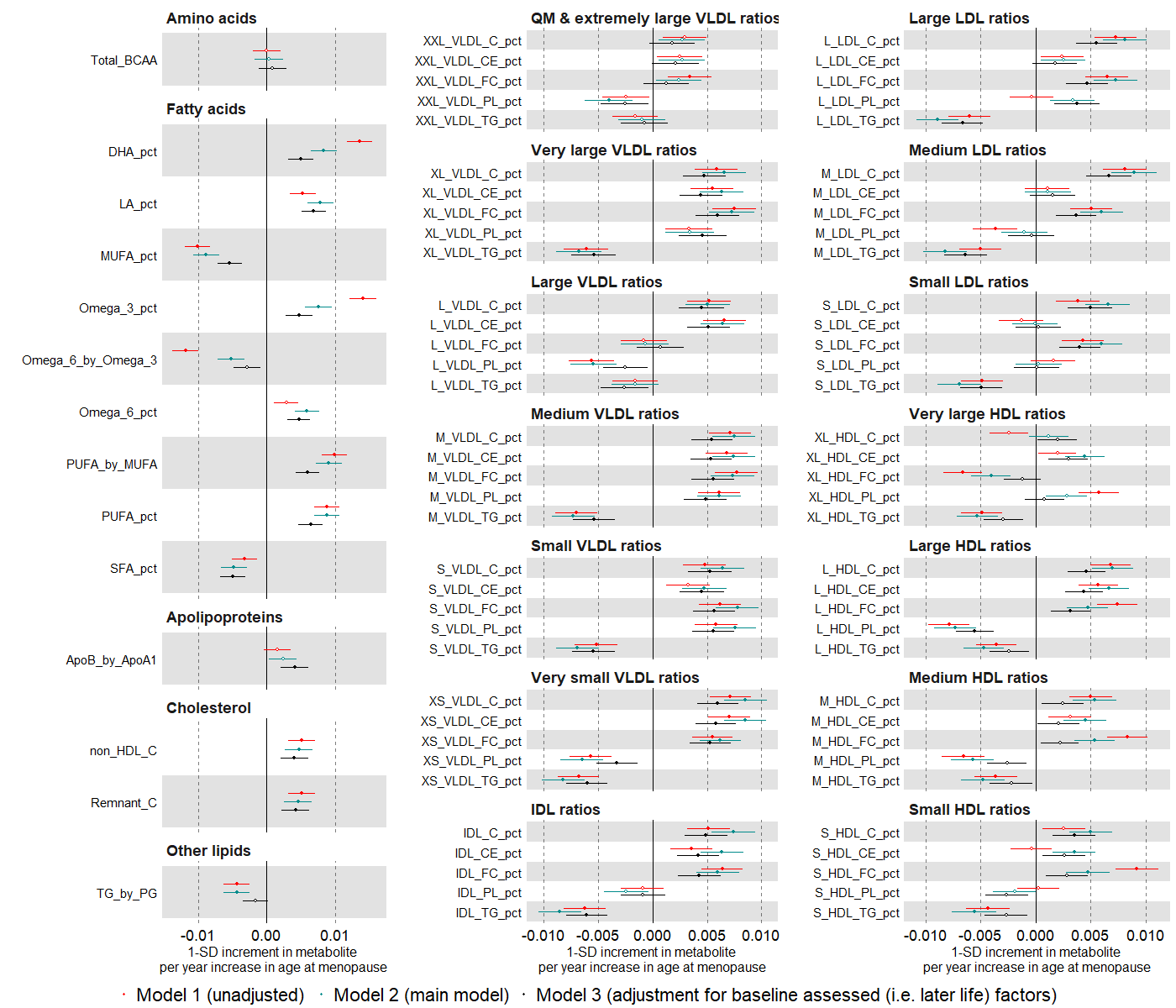


# Suppl Fig 13. Multivariable regression estimates for the relation between age at natural menopause (categorised: <49 (reference), 49-50, 51-53, >53 years) and metabolic measures among females. Footnote: Results are mean differences presented as standard unit changes in metabolic measure for parity 49-50 vs <49, 51-53 vs <49 and >53 vs <49 years, respectively. Circles denote the mean differences and indicate p-value < 0.00093 (filled circles) or ≥ 0.00093 (hollow circles). Horizontal bars denote 95% confidence intervals. Multivariable regression models (ordinary least squares, two-sided regression coefficients reported) were adjusted for age at recruitment, body size at age 10 and education (N=36,253). (a)


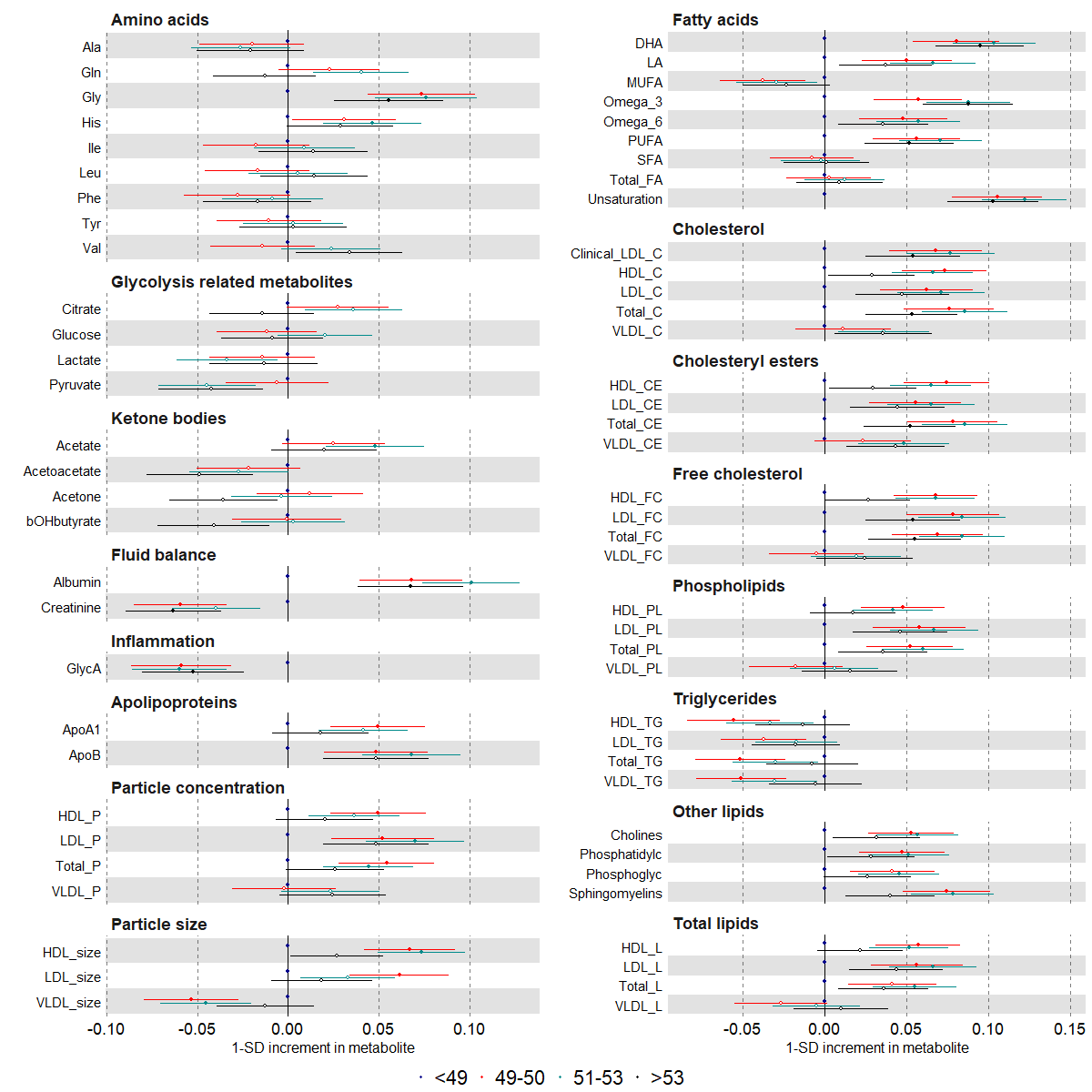


# Suppl Fig 13. (b)


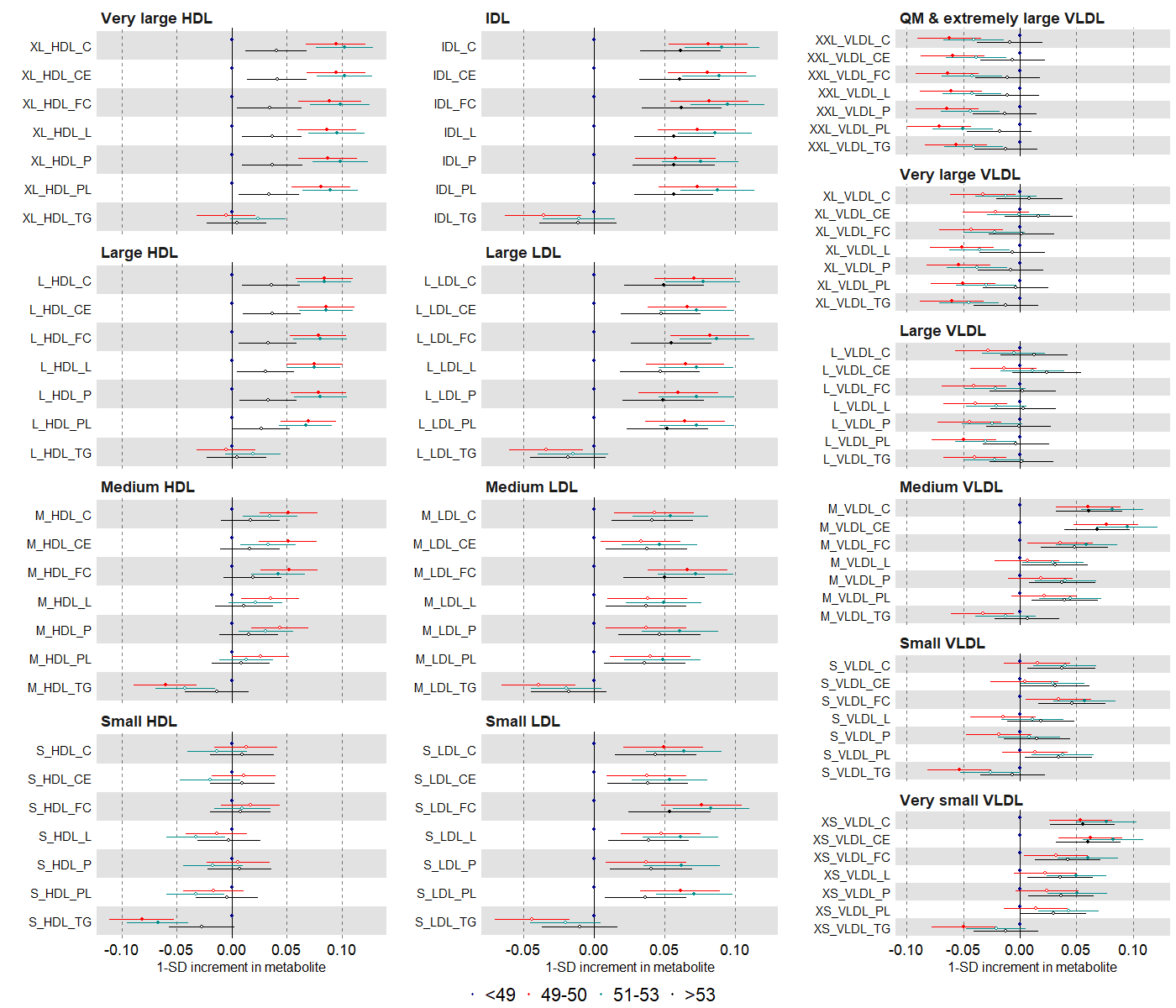


# Suppl Fig 13. (c)


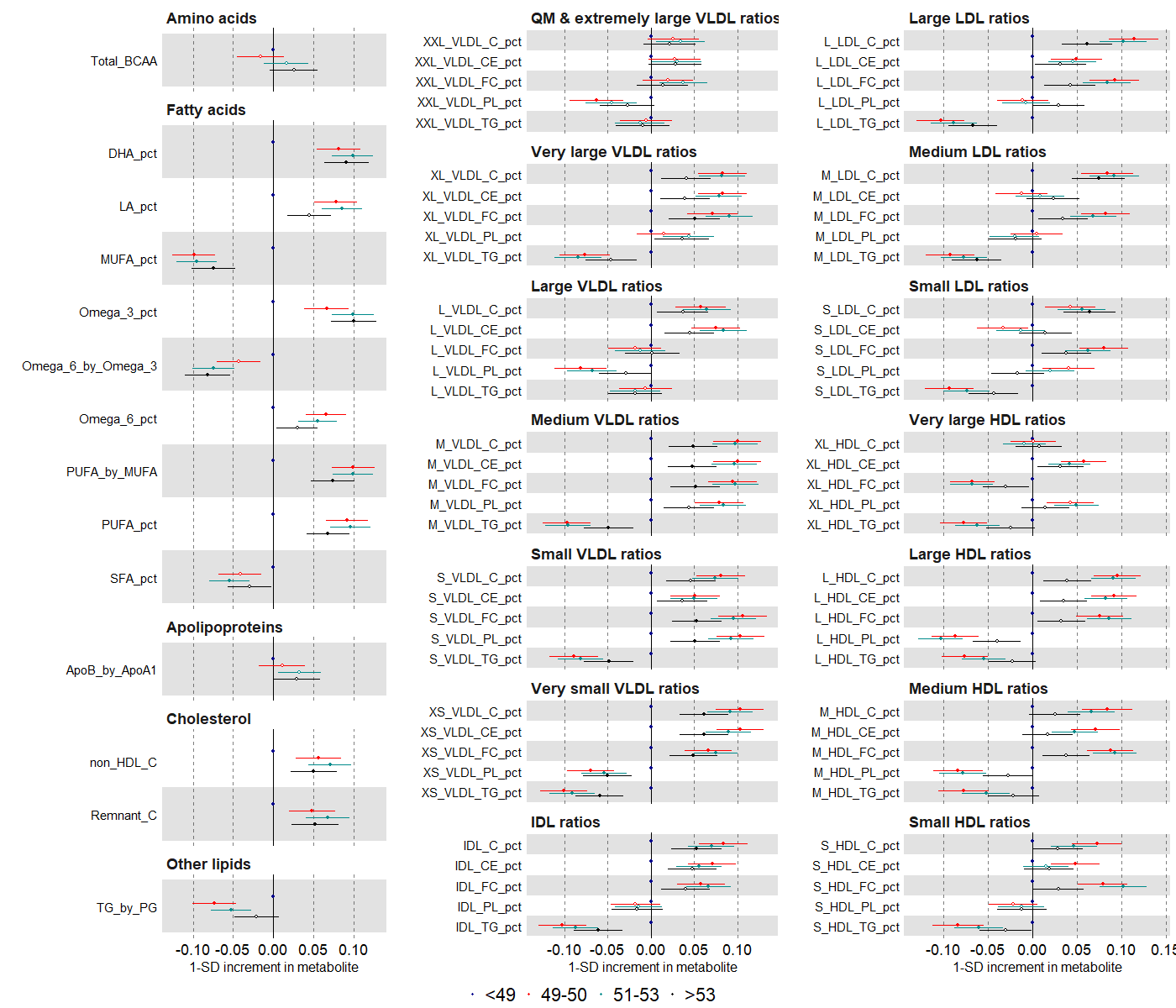


# Suppl Fig 14. Multivariable regression estimates for the relation between age at menopause (restricted cubic splines with knots placed at ages 40, 49, 52, and 56) and metabolic measures among females. Footnote: Mean predicted outcome levels at different menopause ages for a woman who is 60 years old, had an average body size at age 10 and is educated to college or university level (N=36, 253). Error bars denote 95% confidence intervals (a)


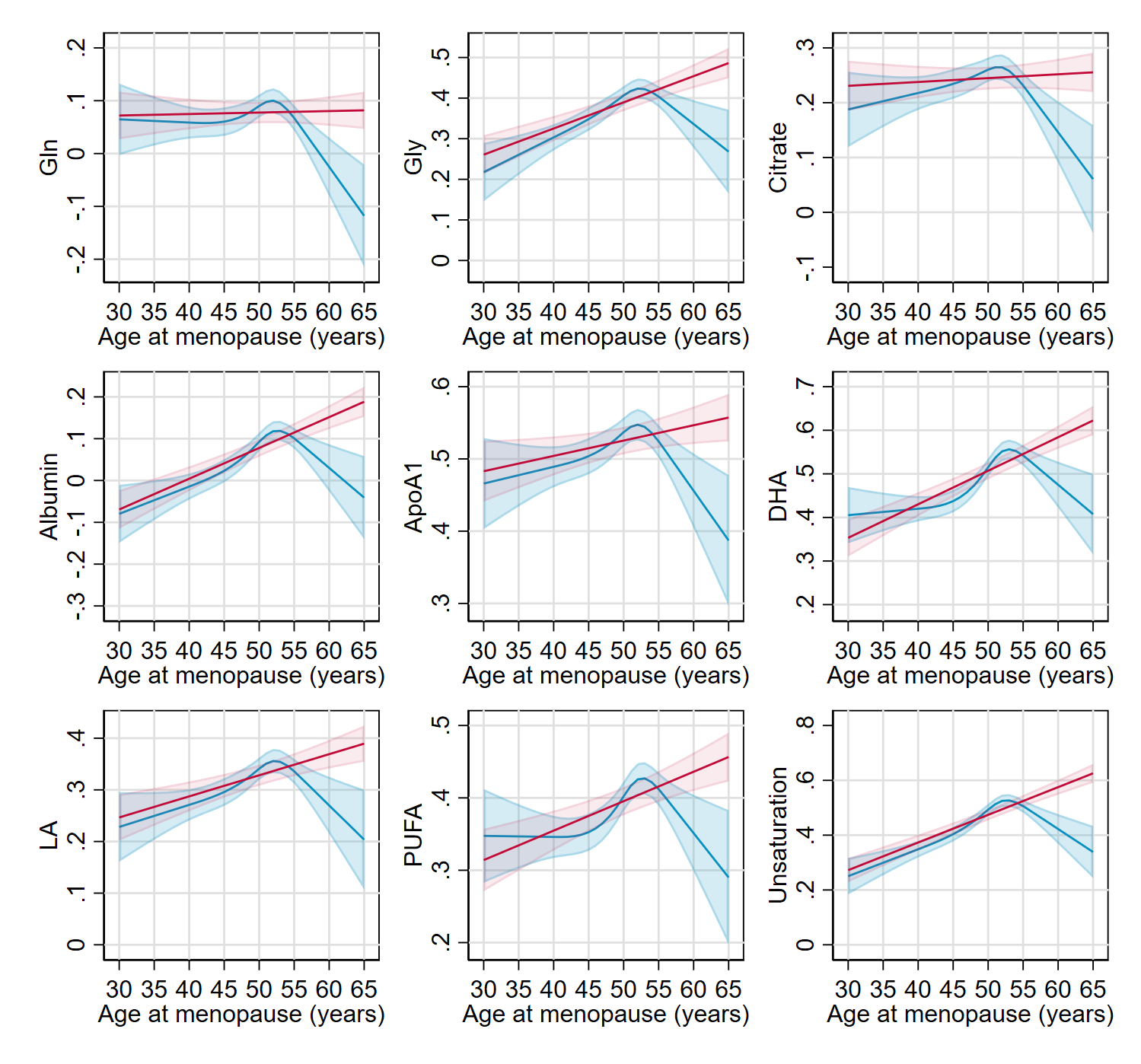


# Suppl Fig 14. (b)


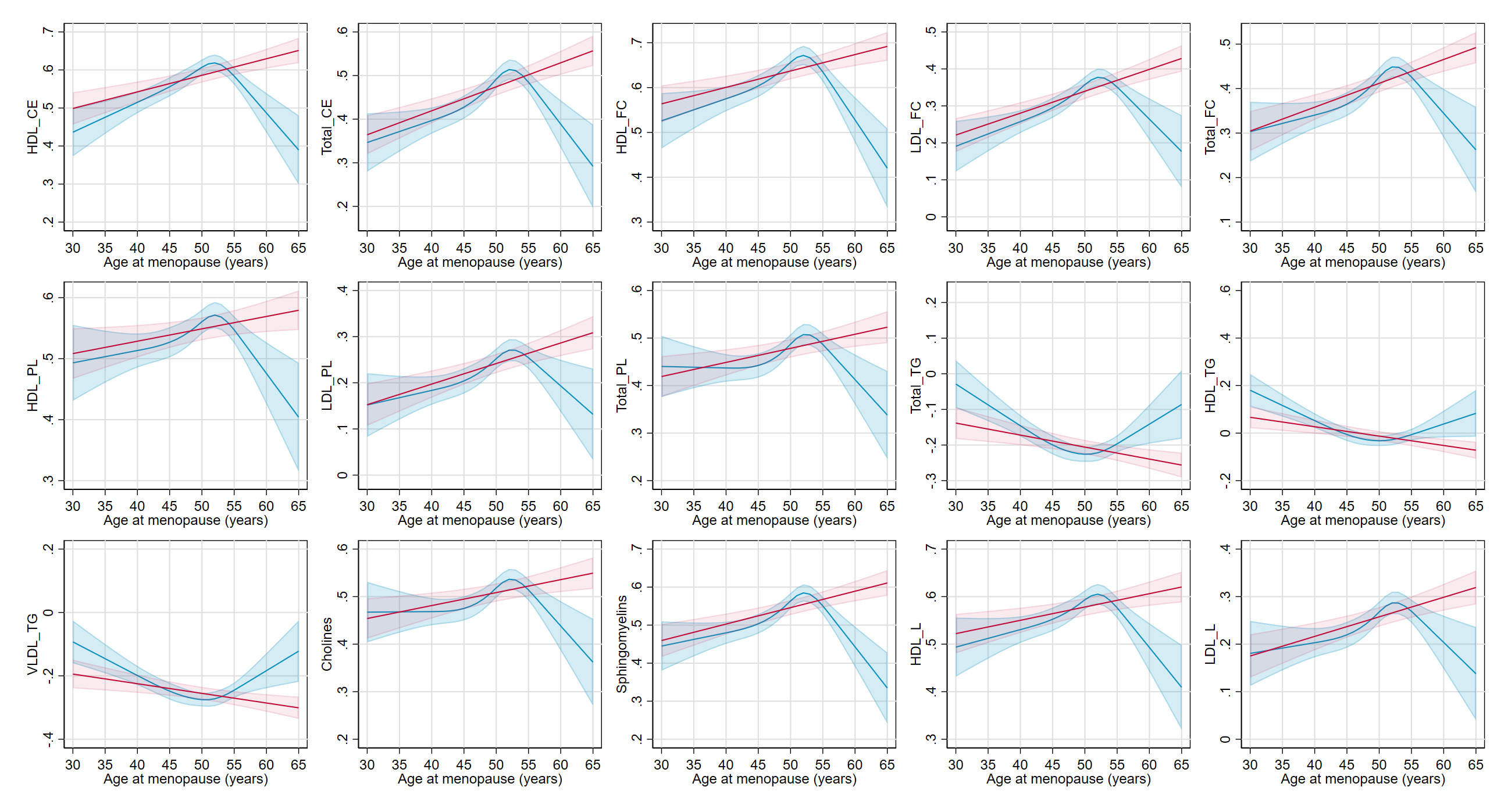


# Suppl Fig 15. Age-combined and age-stratified estimates for the association between age at natural menopause and clinical chemistry biomarkers using multivariable regression (red) and one-sample Mendelian randomization (blue) restricted to women with data on age at menopause (‘selected sample’, N=123,278) or two-sample Mendelian randomization (black) using data from all women (‘full sample’, N= 208,062)


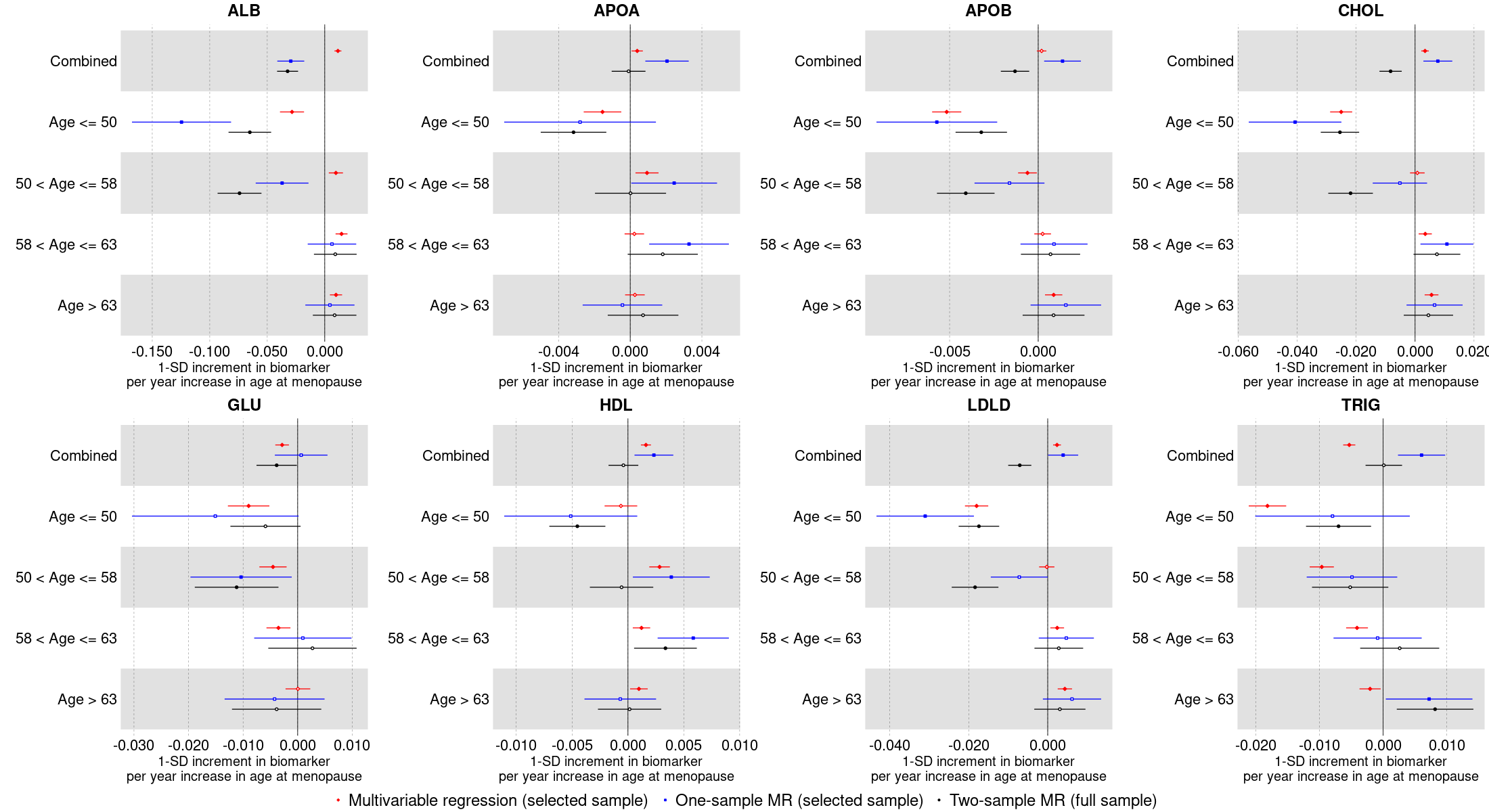


Multivariable regression was performed for women with non-missing data on age at natural menopause using ordinary least squares (OLS) regression. One-sample Mendelian randomization was performed for women with non-missing data on age at natural menopause using two-stage least square (2SLS) regression. Two-sample Mendelian randomization was performed for all women regardless of missing data on age at natural menopause using the inverse variance weighted (IVW) method.

# Suppl Fig 16A. Age-combined and age-stratified estimates for the association between age at natural menopause and clinical chemistry biomarkers excluding users of statins at baseline estimated using multivariable regression (red) and Mendelian randomization (blue) restricted to women with data on age at menopause (‘selected sample’, N=123,278) or Mendelian randomization (black) using data from all women (‘full sample’, N=208,062).


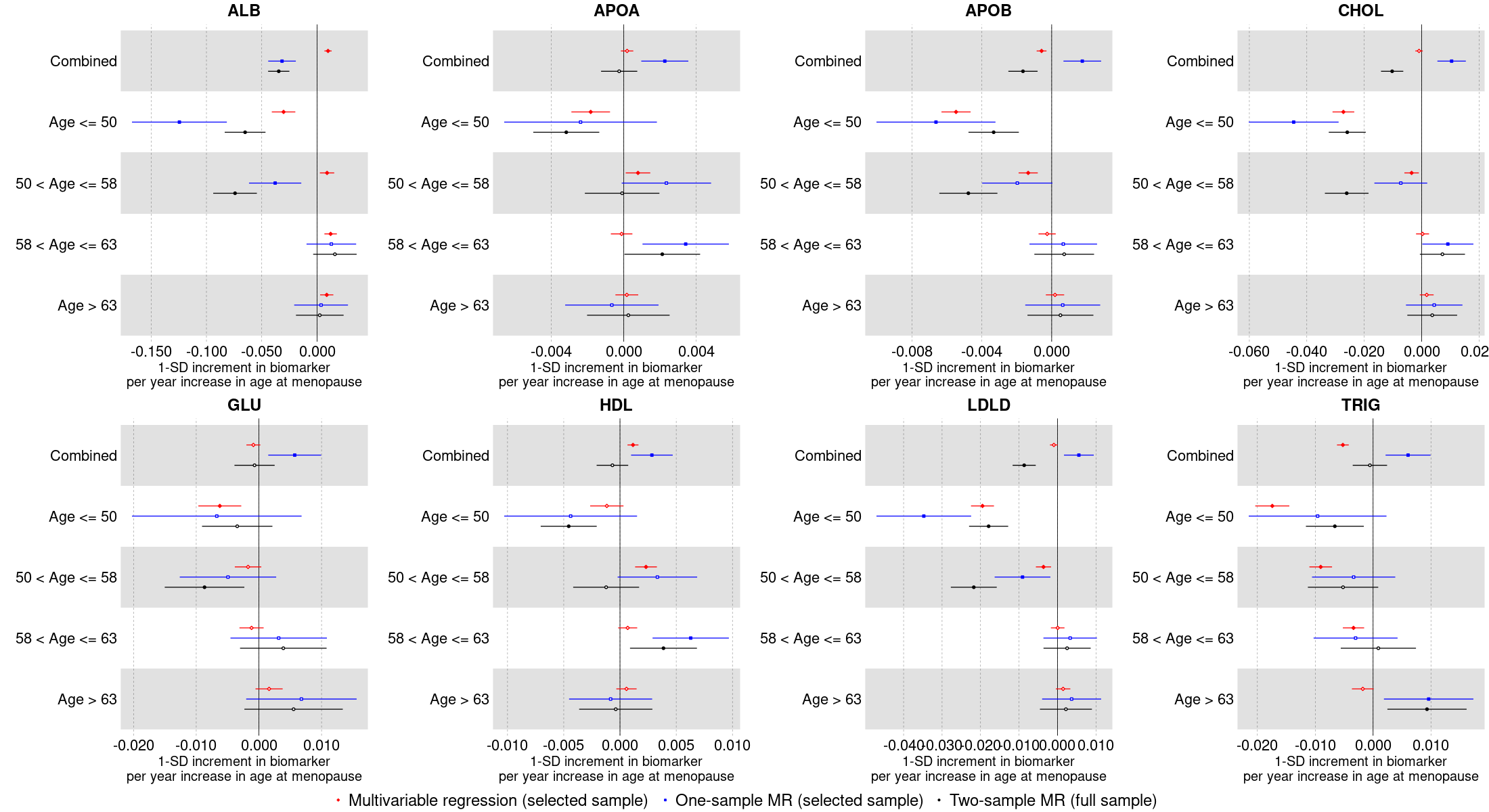


Multivariable regression was performed for women with non-missing data on age at natural menopause using ordinary least squares (OLS) regression. One-sample Mendelian randomization was performed for women with non-missing data on age at natural menopause using two-stage least square (2SLS) regression. Two-sample Mendelian randomization was performed for all women regardless of missing data on age at natural menopause using the inverse variance weighted (IVW) method.

# Suppl Fig 16B. Age-combined and age-stratified estimates for the association between age at natural menopause and clinical chemistry biomarkers excluding users of hormone replacement therapy (HRT) at baseline estimated using multivariable regression (red) and Mendelian randomization (blue) restricted to women with data on age at menopause (‘selected sample’, N=123,278) or Mendelian randomization (black) using data from all women (‘full sample’, N=208,062).


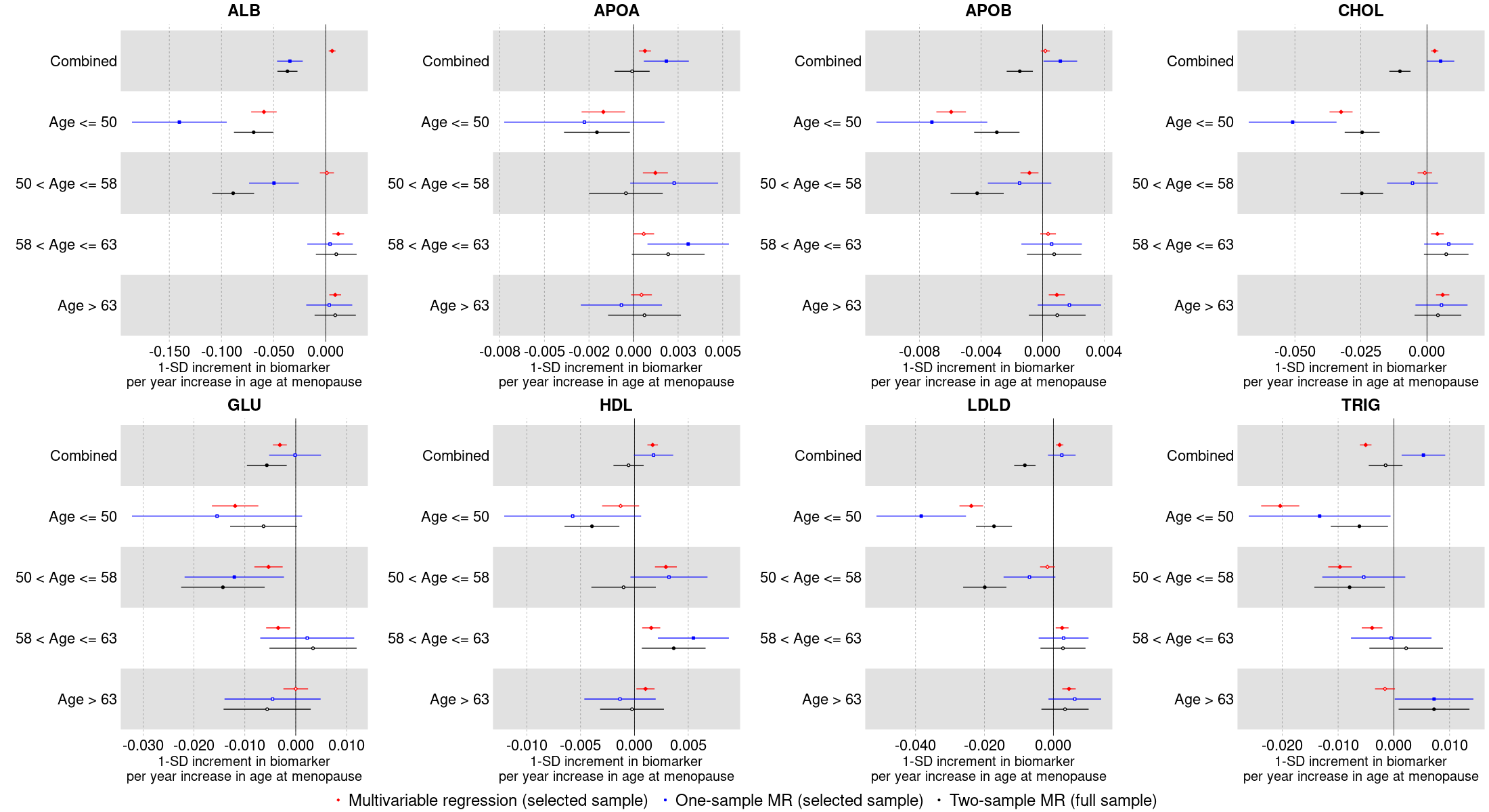


Multivariable regression was performed for women with non-missing data on age at natural menopause using ordinary least squares (OLS) regression. One-sample Mendelian randomization was performed for women with non-missing data on age at natural menopause using two-stage least square (2SLS) regression. Two-sample Mendelian randomization was performed for all women regardless of missing data on age at natural menopause using the inverse variance weighted (IVW) method.

# Suppl Fig 17. Mendelian randomization estimates for the relation between reproductive markers and conventional biomarkers among unrelated individuals and within siblings Footnote: Mendelian randomization models were estimated using the inverse variance weighted method (Within-siblings: N=11,551; 19,312; 4,365 for age at menarche, parity and age at natural menopause and N=26,155 (mean) across biomarkers. Unrelated: N=27,880; 58,128; 11,611 for age at menarche, parity and age at natural menopause and N=64,823 (mean) across biomarkers).


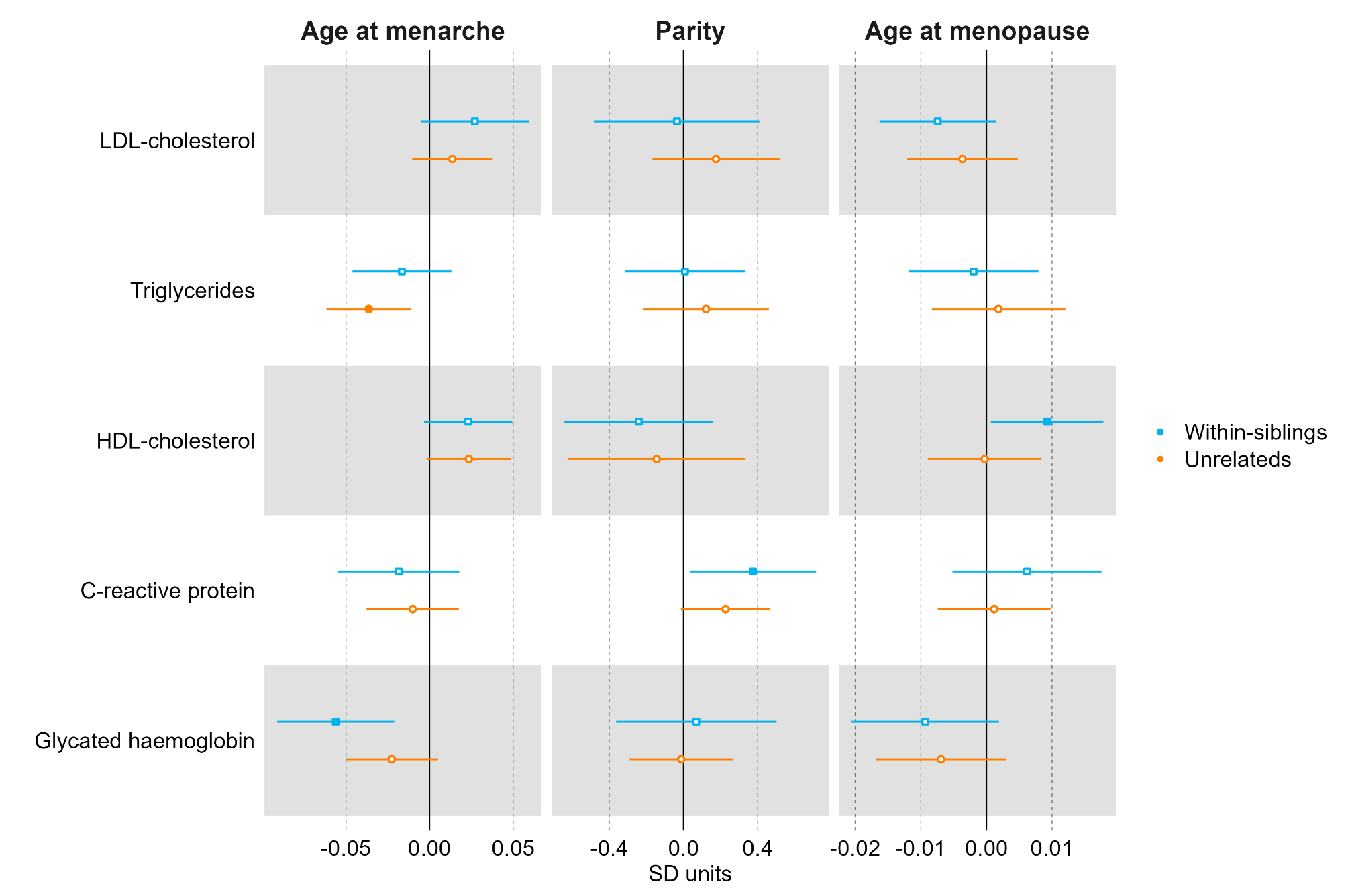


# Suppl Fig 18. Mendelian randomization estimates for the relation between older age at menarche and metabolic measures among females (comparing different Mendelian randomization methods). Footnote: Mendelian randomization models were estimated using the inverse variance weighted method (N= 62,209) (a)


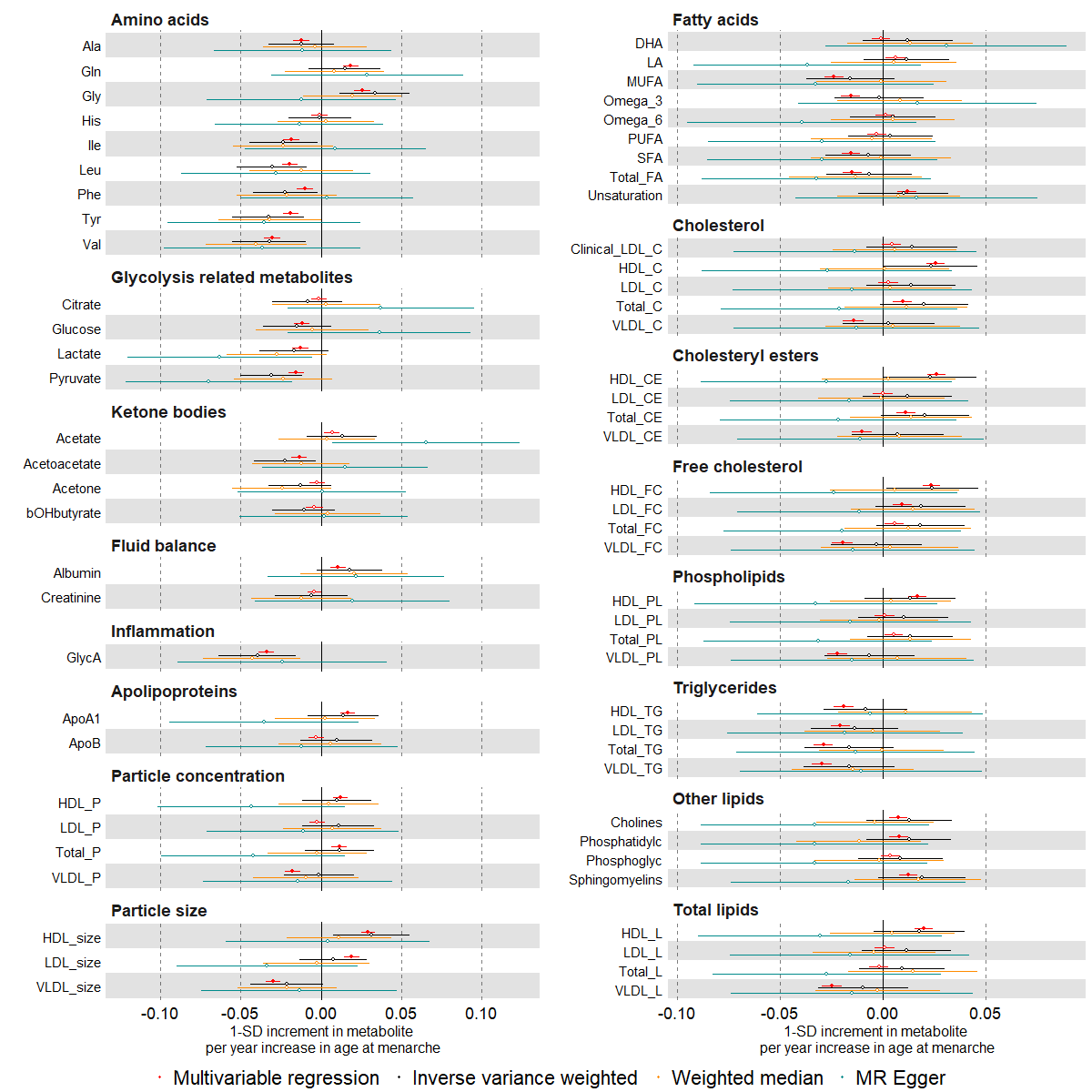


# Suppl Fig 18. (b)


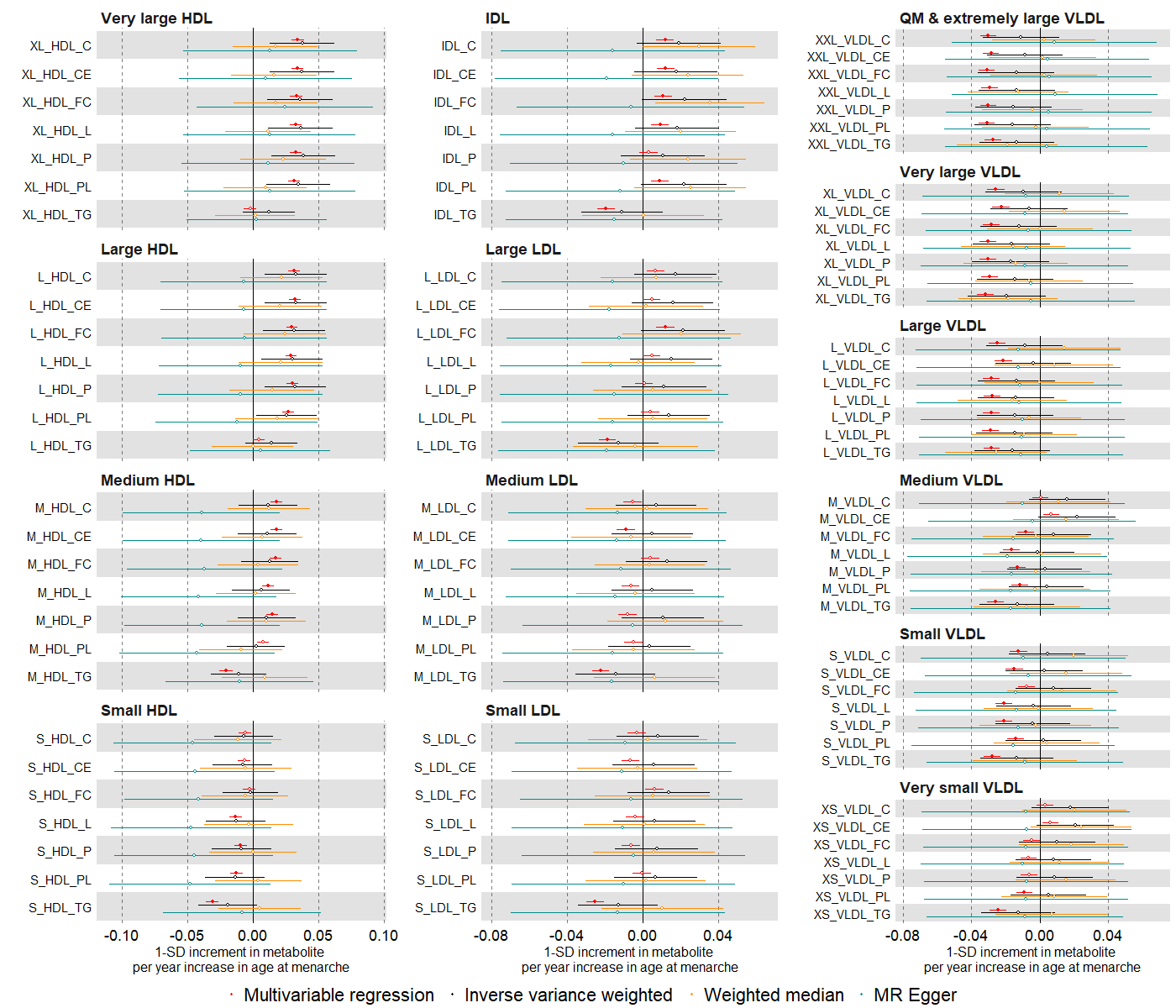


# Suppl Fig 18. (c)


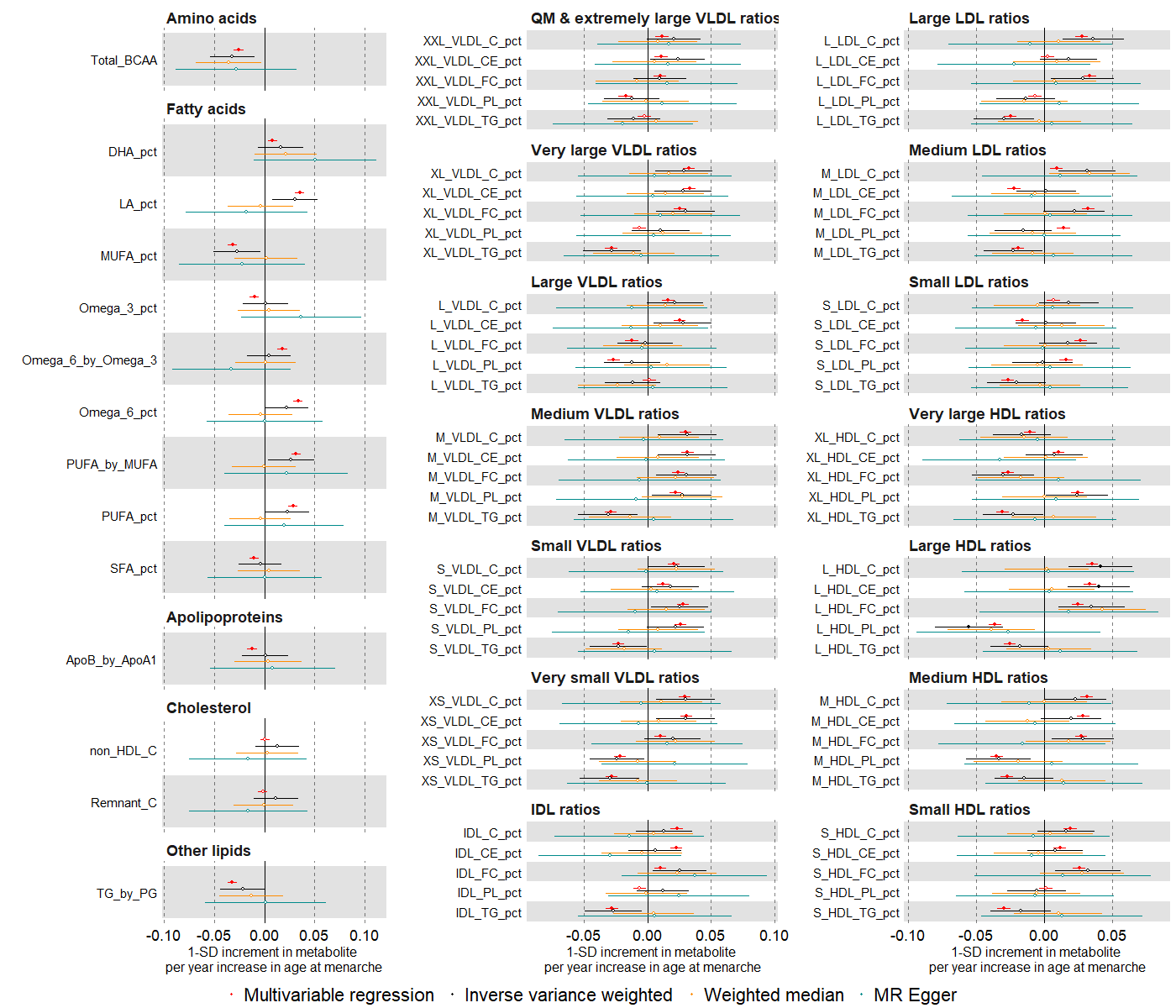


# Suppl Fig 19. Mendelian randomization estimates for the relation between higher parity and metabolic measures among females (comparing different Mendelian randomization methods). Mendelian randomization models were estimated using the inverse variance weighted method (N= 62,209) (a)


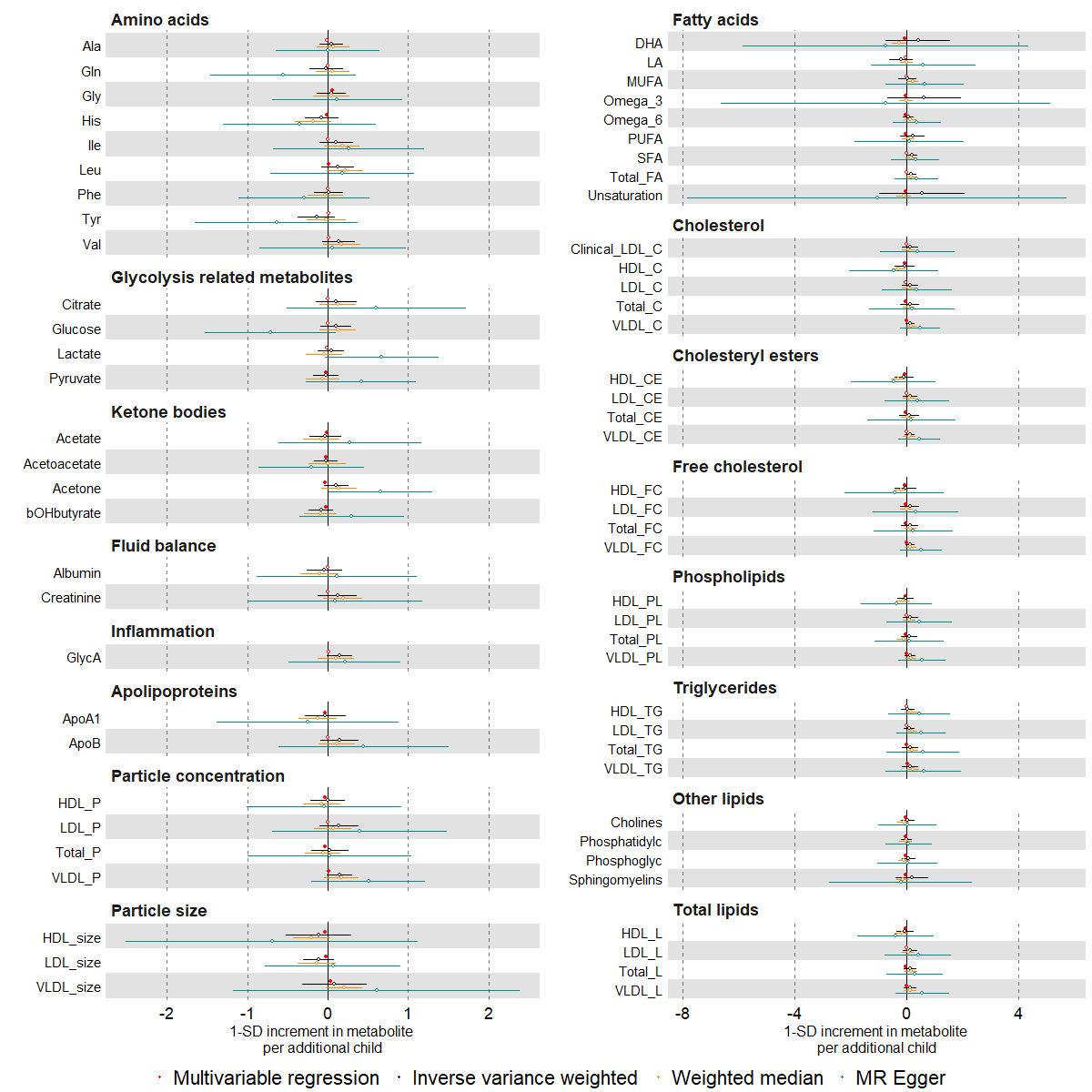


# Suppl Fig 19. (b)


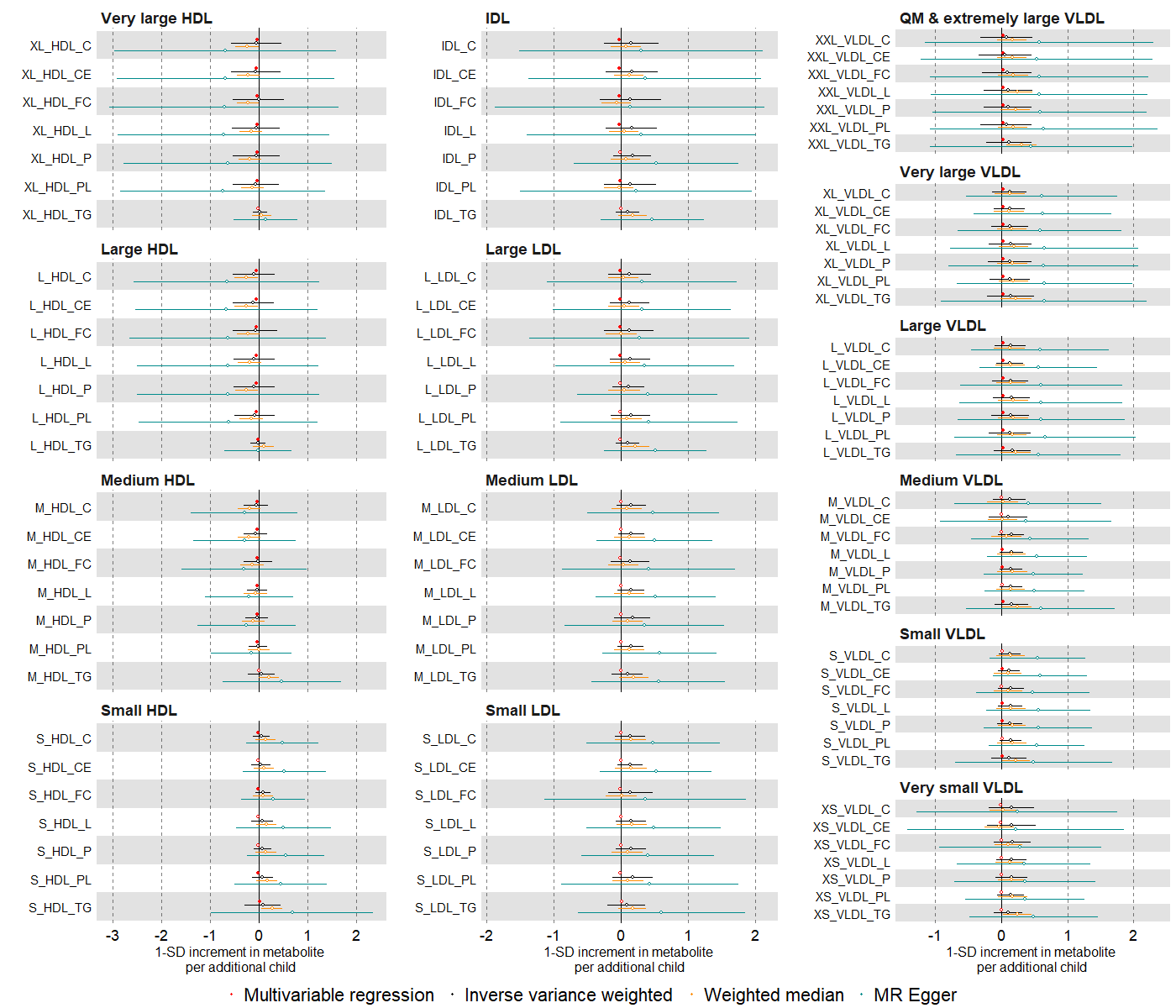


# Suppl Fig 19. (c)


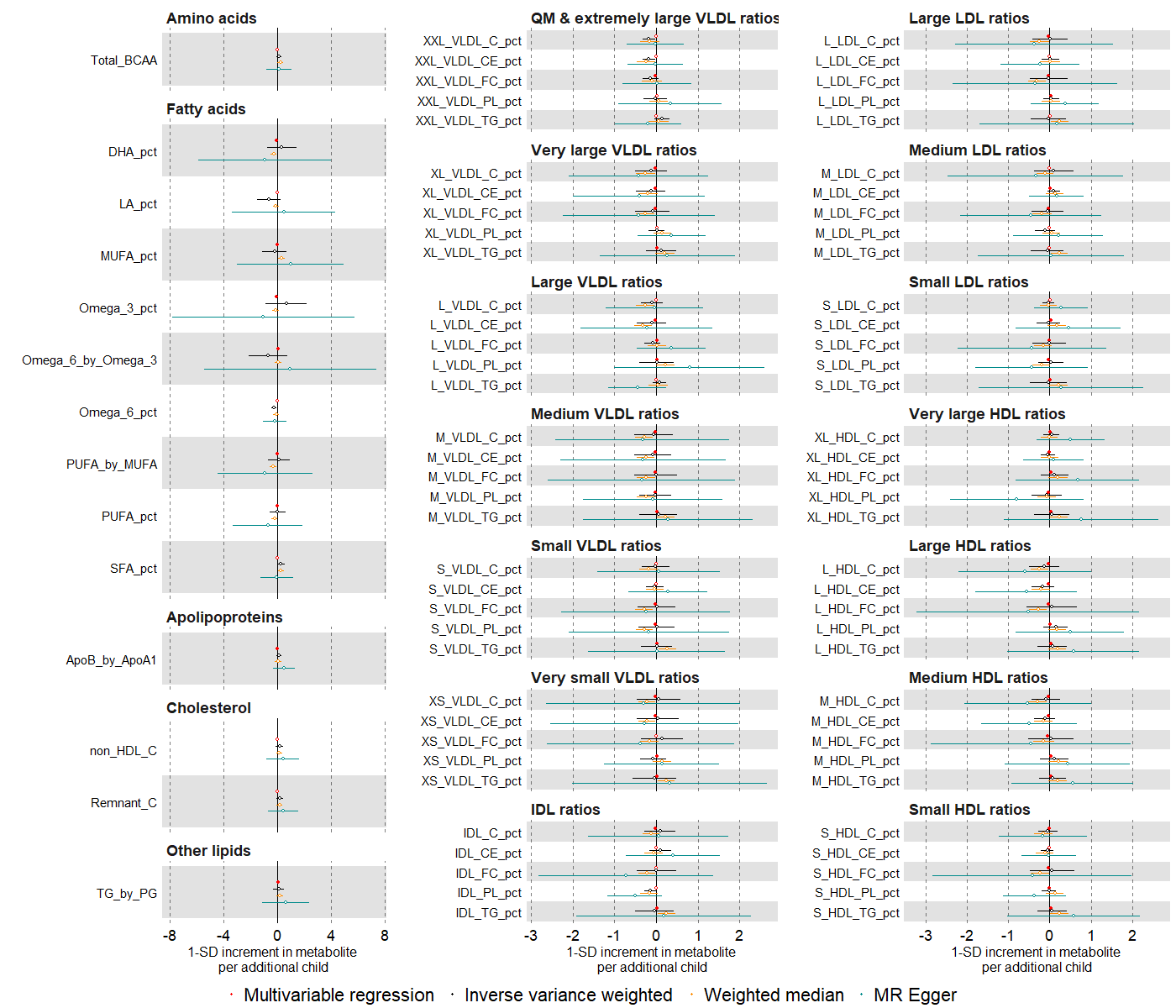


# Suppl Fig 20. Mendelian randomization estimates for the relation between older age at natural menopause and metabolic measures among females (compare different Mendelian randomization methods). Mendelian randomization models were estimated using the inverse variance weighted method (N= 62,209) (a)


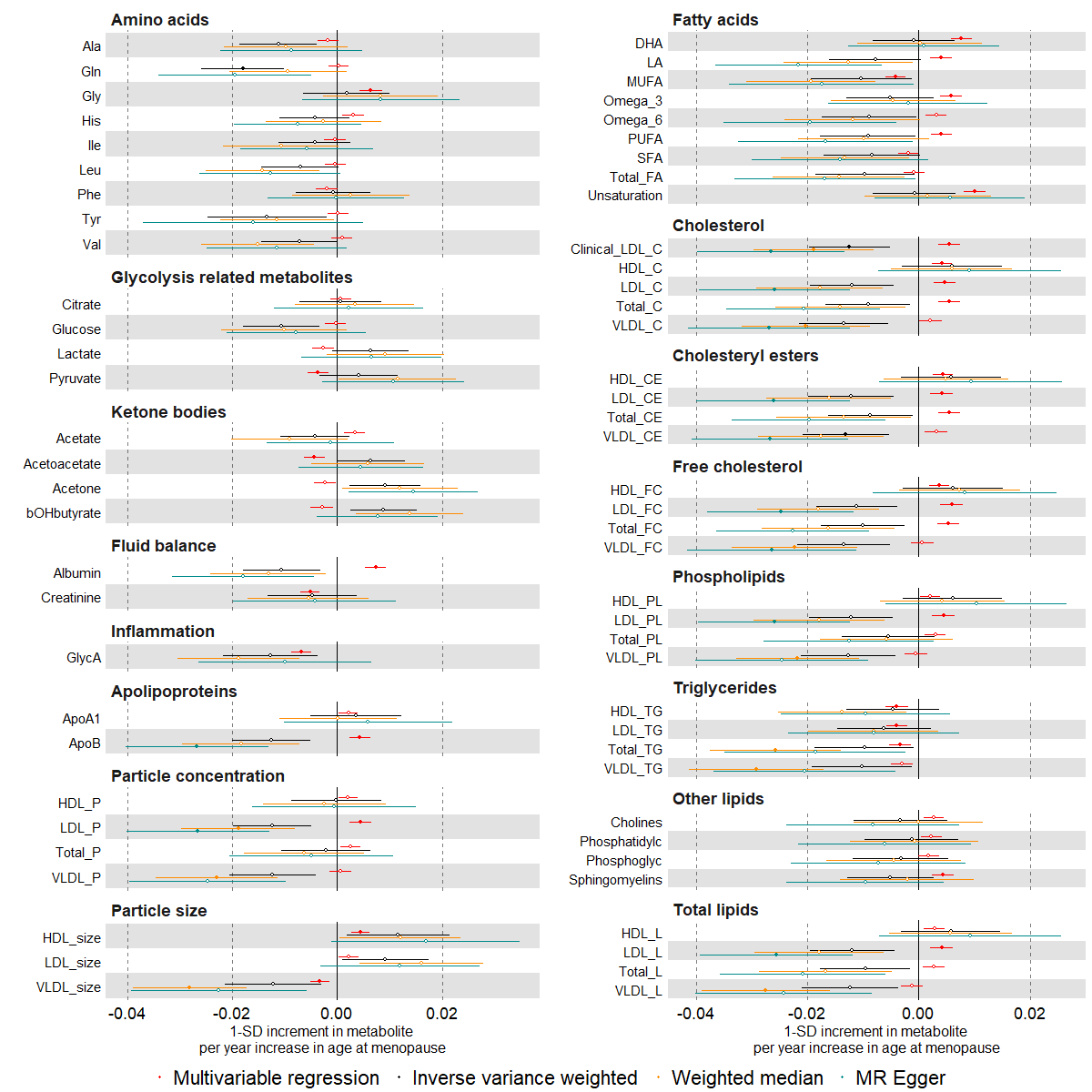


# Suppl Fig 20. (b)


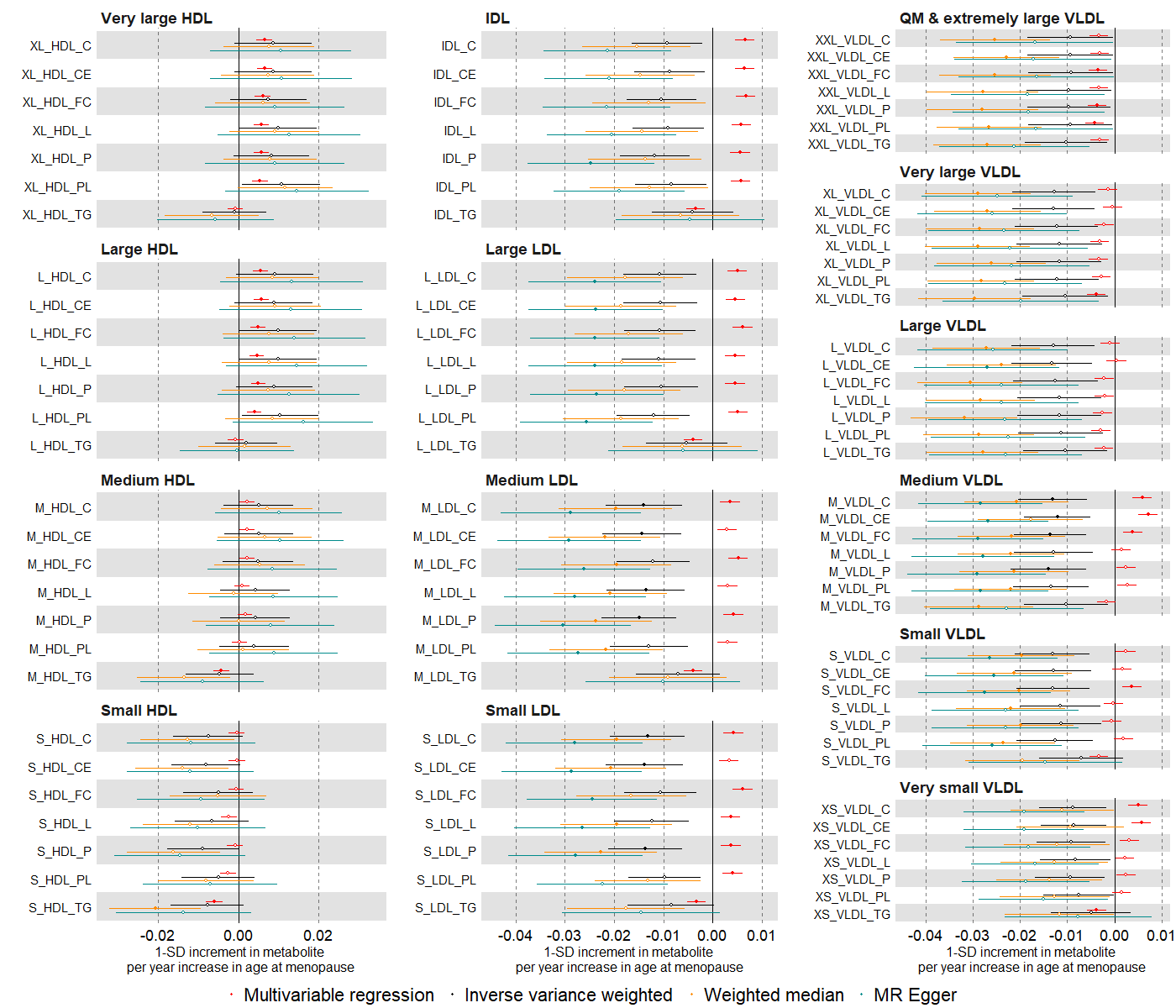


# Suppl Fig 20. (c)


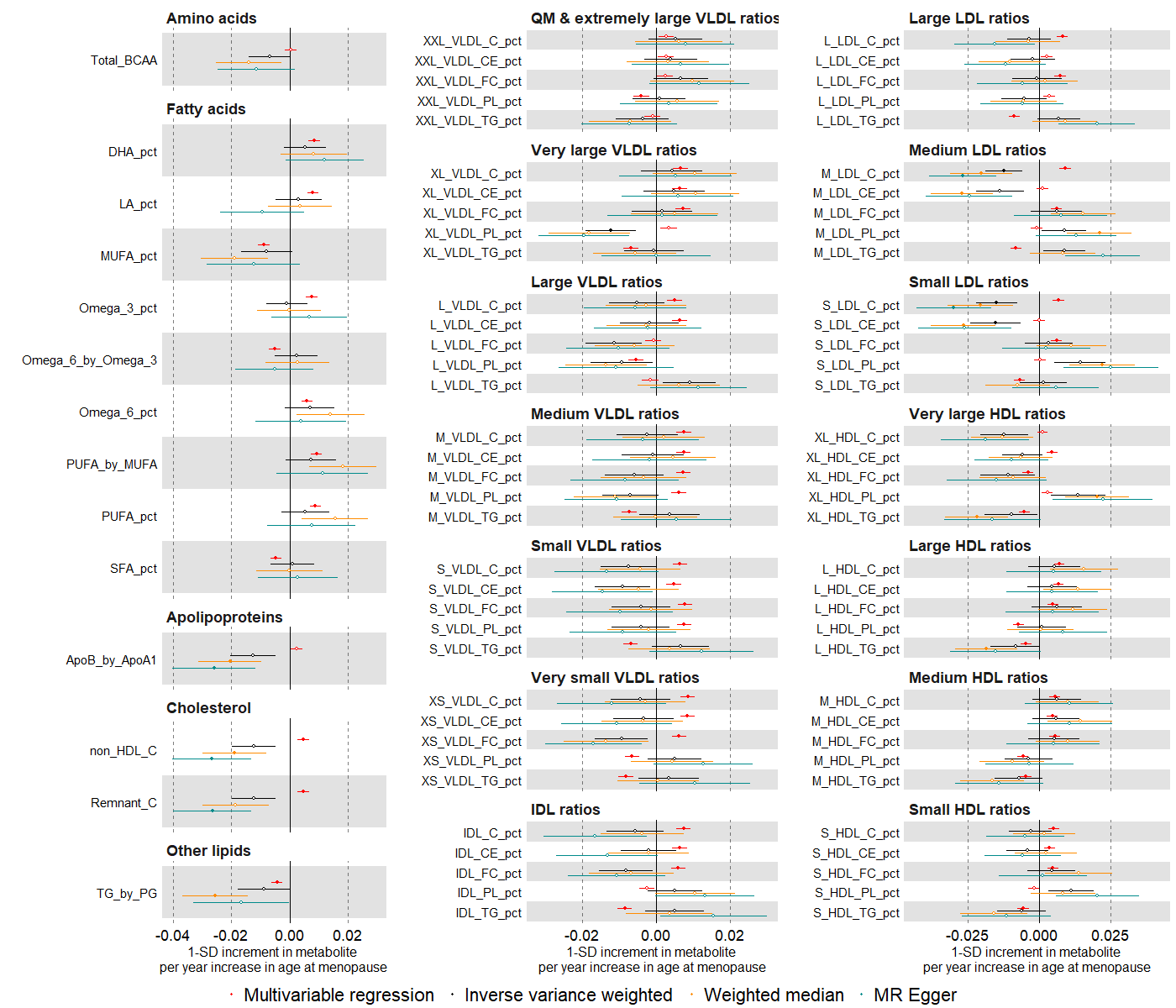


# Suppl Fig 21. Mendelian randomization estimates for the relation between older age at menarche and metabolic measures among females (comparing different SNP sets). Mendelian randomization models were estimated using the inverse variance weighted method (N= 62,209) (a)


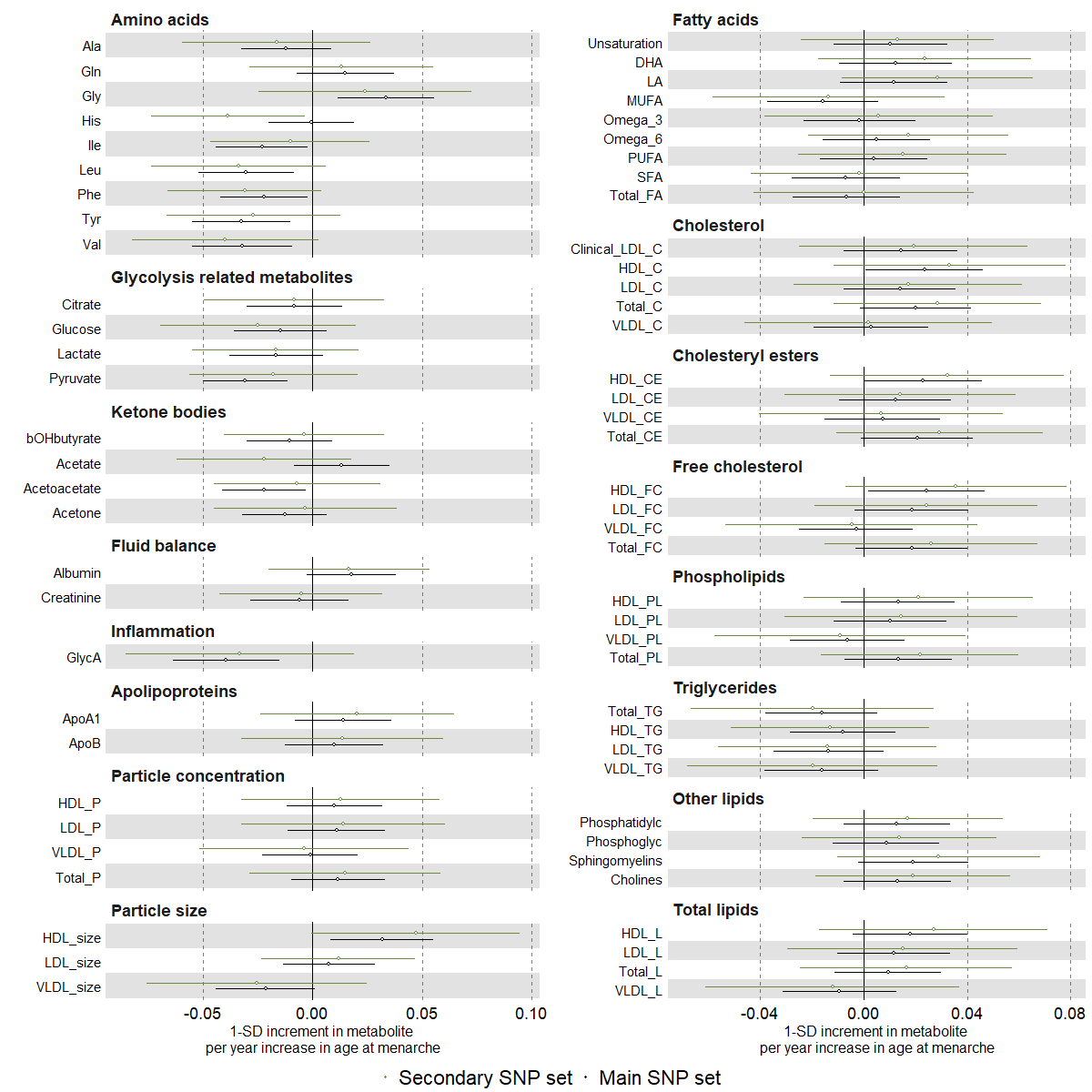


# Suppl Fig 21. (b)


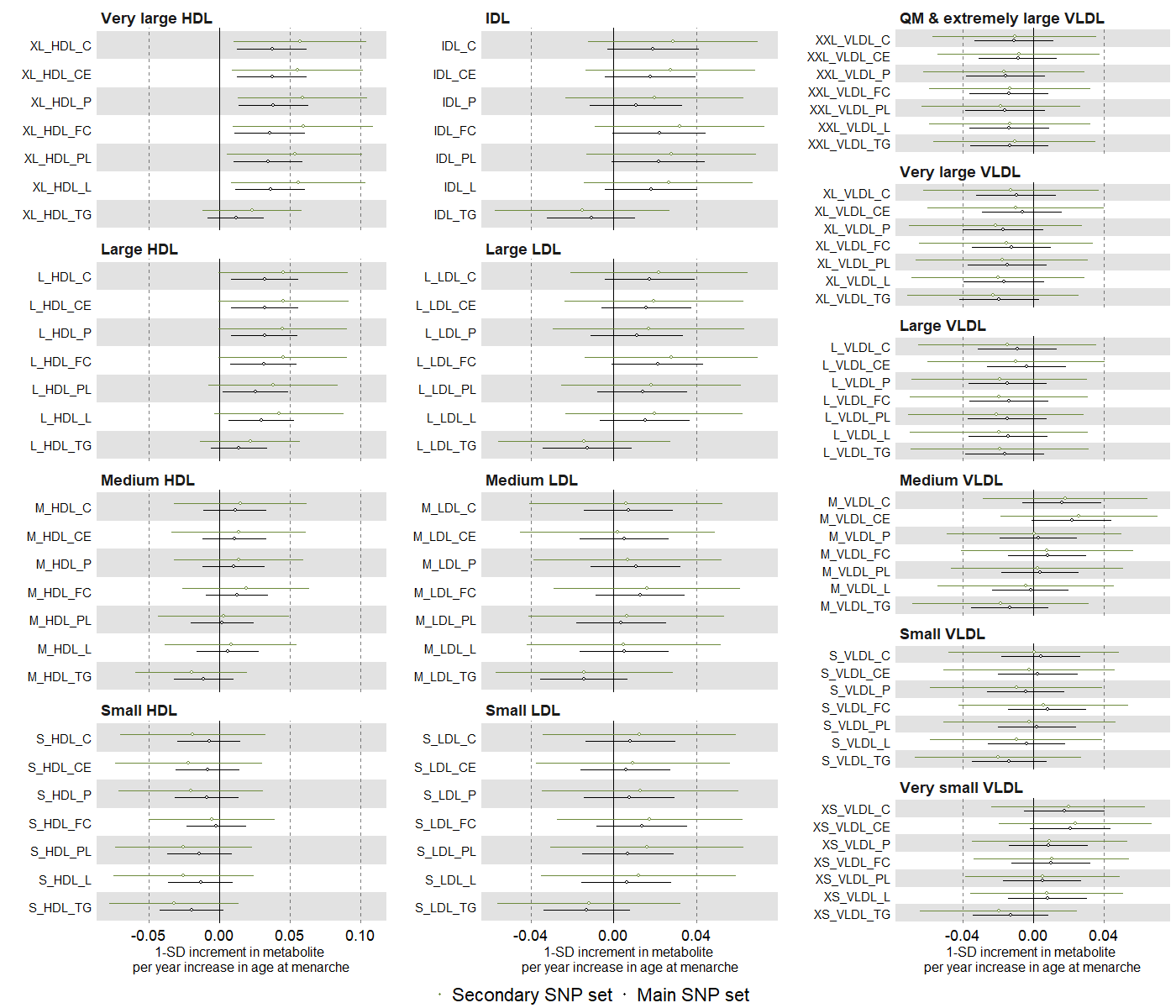


# Suppl Fig 21. (c)


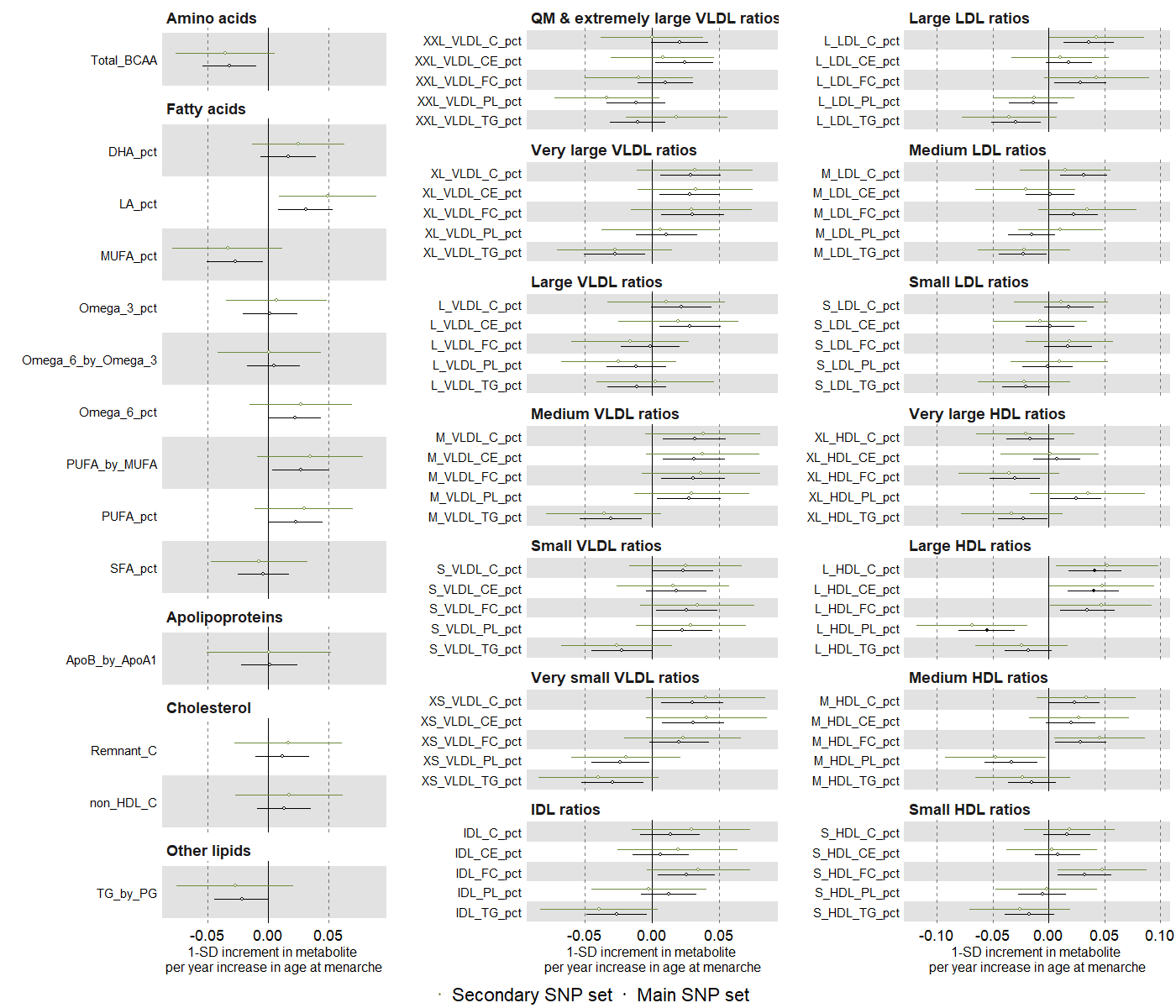


# Suppl Fig 22. Mendelian randomization estimates for the relation between older age at natural menopause and metabolic measures among females (comparing different SNP sets). Mendelian randomization models were estimated using the inverse variance weighted method (N= 62,209) (a)


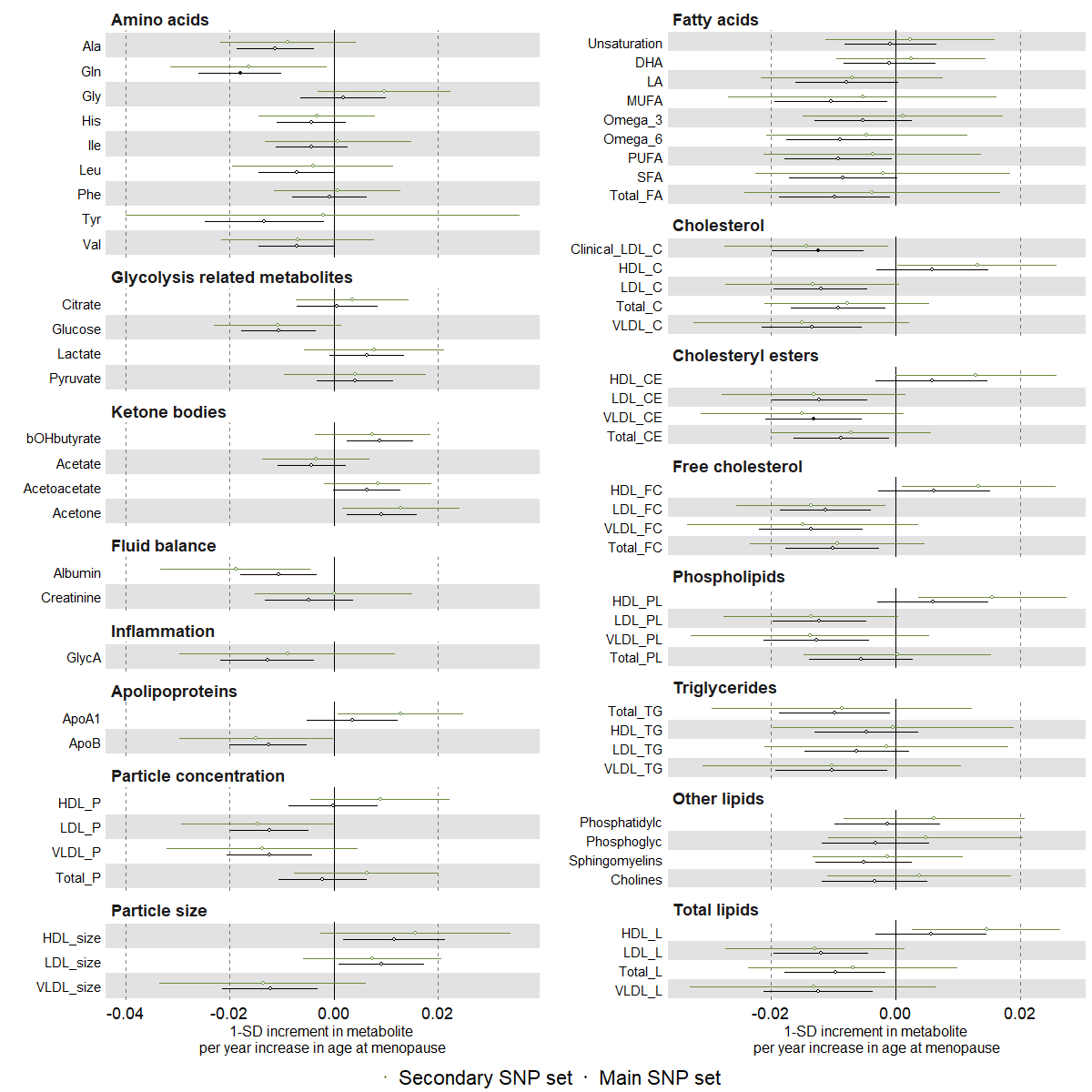


# Suppl Fig 22. (b)


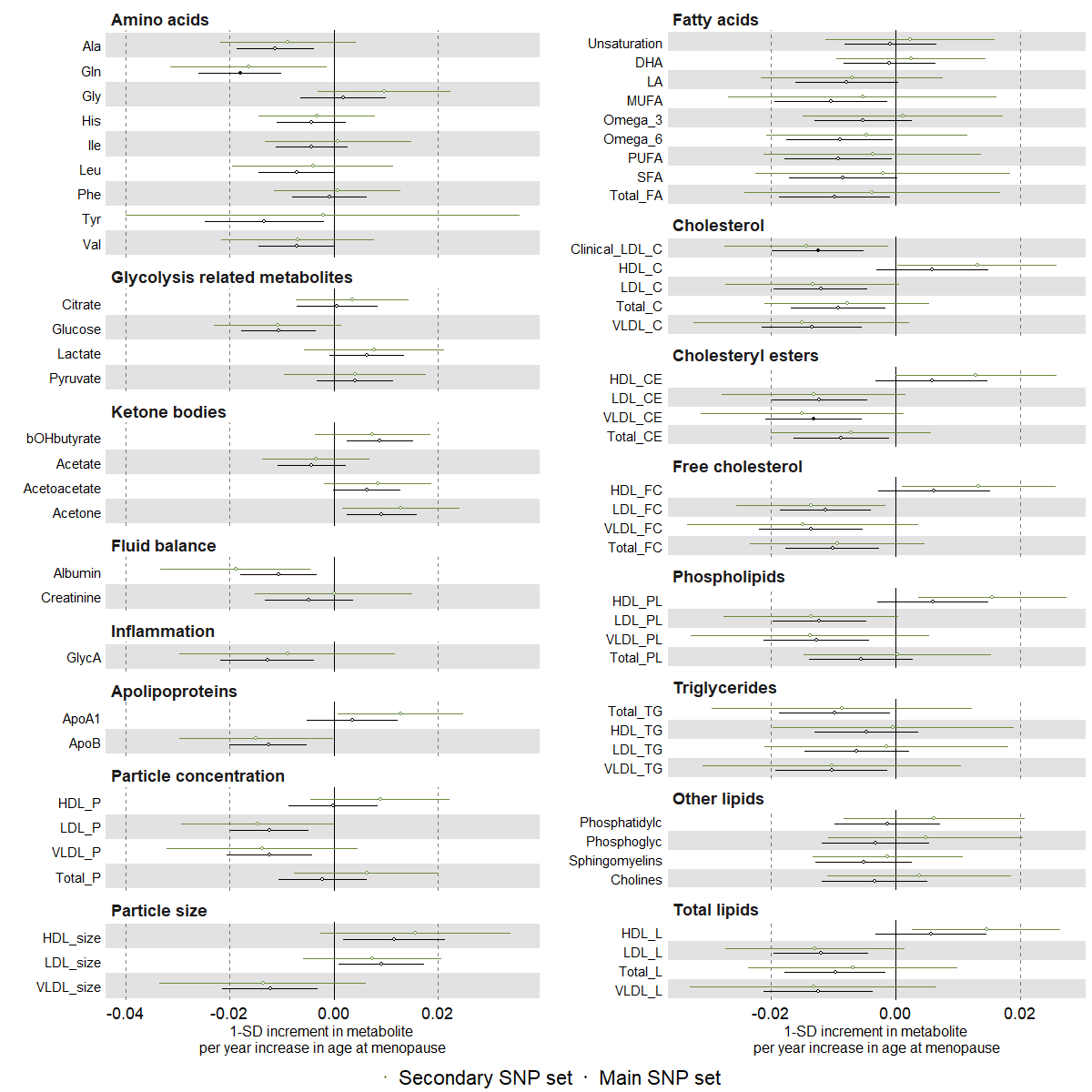


# Suppl Fig 22. (c)


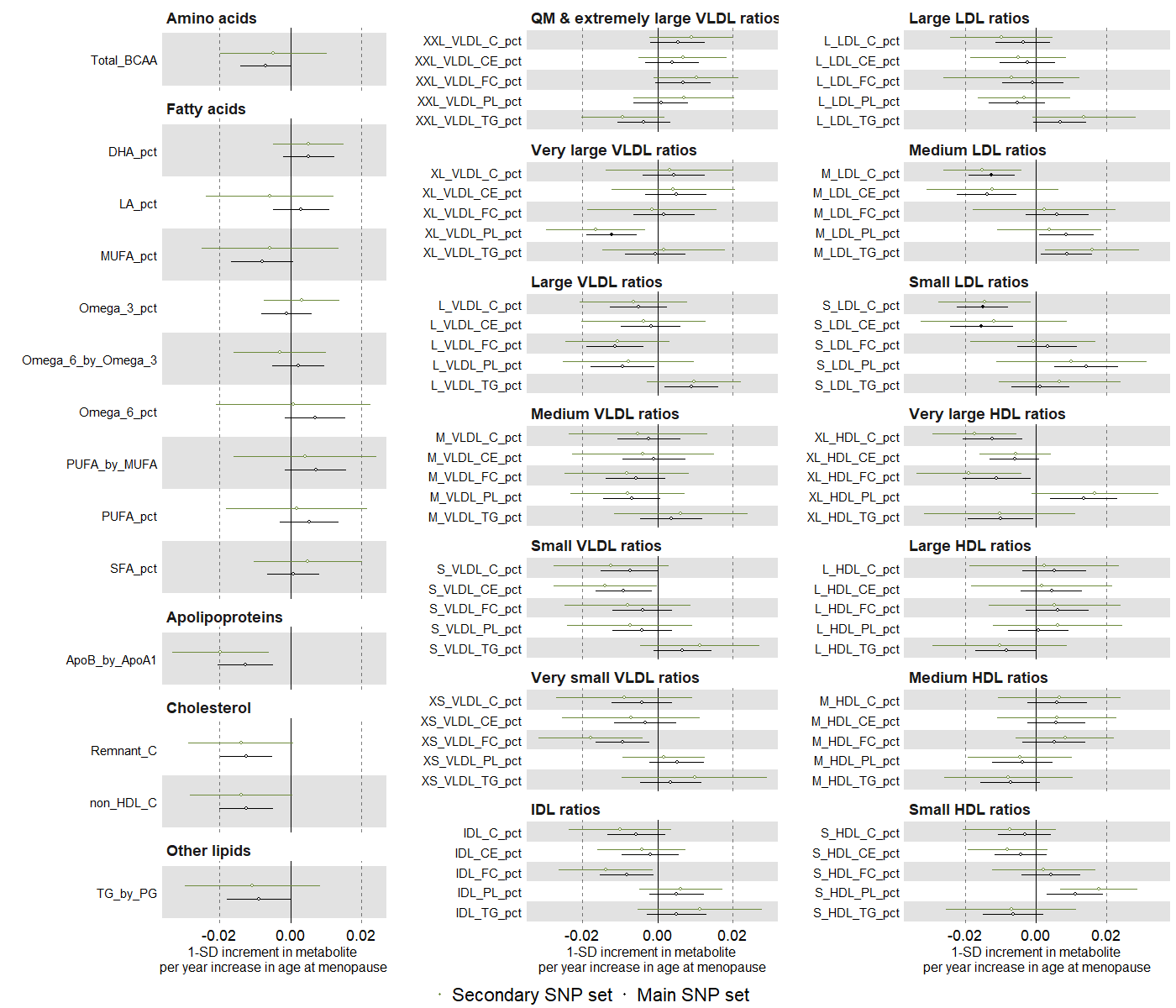

Supplement: Supplementary file 1 — Supplementary figures [file 41467_2023_44459_MOESM1_ESM.docx]
