# Supplementary material for: The impact of reproductive factors on the metabolic profile of females from menarche to menopause
Source: Nat Commun. 2024 Feb 6;15:1103. doi: 10.1038/s41467-023-44459-6 (PMC10847109; doi:10.1038/s41467-023-44459-6)
Supplement: Supplementary file 3 — Description of Additional Supplementary Files [file 41467_2023_44459_MOESM3_ESM.pdf]

Description of Additional Supplementary Files

Supplementary Datasets

Supplementary Data 1 - Distribution of characteristics of UK Biobank participants females by categories of age at menarche (<13, 13-14, >14 years)

Supplementary Data 2 - Distribution of characteristics of UK Biobank participants females and males by categories of parity/number of children (0, 1, 2, 3+)

Supplementary Data 3 - Distribution of characteristics of UK Biobank females with NMR metabolomics data by categories of menopausal status

Supplementary Data 4 - Distribution of metabolic measures among UK Biobank females

Supplementary Data 5 - Multivariable regression and mendelian randomisation estimates for the associations between age at menarche and metabolic measures

Supplementary Data 6 - P-value for non linear test for trend for each reproductive trait

Supplementary Data 7 - Model fit statistics between linear model and restricted cubic splines models (varying number of knots) for age at menarche

Supplementary Data 8 - Characteristics of genome-wide association studies used to select genetic instruments for reproductive traits

Supplementary Data 9 - Multivariable regression, paternal negative control and mendelian randomisation estimates for the associations between parity (number of children) and metabolic measures

Supplementary Data 10 - Model fit statistics between linear model and restricted cubic splines models (varying number of knots) for parity

Supplementary Data 11 - Multivariable regression and mendelian randomisation estimates for the associations between age at menopause and metabolic measures

Supplementary Data 12 - Model fit statistics between linear model and restricted cubic splines models (varying number of knots) for age at menopause

Supplementary Data 13 - Mendelian randomization exploring the relation of reproductive traits with negative control outcomes

Supplementary Data 14 - Characteristics of genetic instruments selected for reproductive traits
