# Supplementary material for: The impact of reproductive factors on the metabolic profile of females from menarche to menopause
Source: Nat Commun. 2024 Feb 6;15:1103. doi: 10.1038/s41467-023-44459-6 (PMC10847109; doi:10.1038/s41467-023-44459-6)
Supplement: Supplementary file 5 — Reporting Summary [file 41467_2023_44459_MOESM5_ESM.pdf]

nature portfolio

Corresponding author(s): Gemma L Clayton (corresponding author), Maria Carolina Borges, Deborah A Lawlor

Last updated by author(s): 27/07/2023

Reporting Summary

Nature Portfolio wishes to improve the reproducibility of the work that we publish. This form provides structure for consistency and transparency in reporting. For further information on Nature Portfolio policies, see our Editorial Policies and the Editorial Policy Checklist.

Statistics

For all statistical analyses, confirm that the following items are present in the figure legend, table legend, main text, or Methods section.

n/a	Confirmed	
☐	☒	The exact sample size (n) for each experimental group/condition, given as a discrete number and unit of measurement
☐	☒	A statement on whether measurements were taken from distinct samples or whether the same sample was measured repeatedly
☐	☒	The statistical test(s) used AND whether they are one- or two-sided<br>*Only common tests should be described solely by name; describe more complex techniques in the Methods section.*
☐	☒	A description of all covariates tested
☐	☒	A description of any assumptions or corrections, such as tests of normality and adjustment for multiple comparisons
☐	☒	A full description of the statistical parameters including central tendency (e.g. means) or other basic estimates (e.g. regression coefficient) AND variation (e.g. standard deviation) or associated estimates of uncertainty (e.g. confidence intervals)
☐	☒	For null hypothesis testing, the test statistic (e.g. F, t, r) with confidence intervals, effect sizes, degrees of freedom and P value noted<br>*Give P values as exact values whenever suitable.*
☒	☐	For Bayesian analysis, information on the choice of priors and Markov chain Monte Carlo settings
☒	☐	For hierarchical and complex designs, identification of the appropriate level for tests and full reporting of outcomes
☐	☒	Estimates of effect sizes (e.g. Cohen's d, Pearson's r), indicating how they were calculated

Our web collection on statistics for biologists contains articles on many of the points above.

Software and code

Policy information about availability of computer code

Data collection	Data collection was carried out by UK Biobank and Nightingale Ltd. Please refer to "https://biobank.ctsu.ox.ac.uk/crystal/crystal/docs/nmrm_companion_doc.pdf" for all steps during the data collection.
Data analysis	All analysis was conducted using Stata16 (StataCorp, College Station, TX) and R 4.1.1 (R Foundation for Statistical Computing, Vienna, Austria). Analysis scripts and the analysis plan can be found on the following GitHub page: https://github.com/gc13313/nmr_repro.

For manuscripts utilizing custom algorithms or software that are central to the research but not yet described in published literature, software must be made available to editors and reviewers. We strongly encourage code deposition in a community repository (e.g. GitHub). See the Nature Portfolio guidelines for submitting code & software for further information.

Data

Policy information about availability of data

All manuscripts must include a data availability statement. This statement should provide the following information, where applicable:

- Accession codes, unique identifiers, or web links for publicly available datasets
- A description of any restrictions on data availability
- For clinical datasets or third party data, please ensure that the statement adheres to our policy

UK Biobank received ethical approval from the Research Ethics Committee (REC reference 582 for UK Biobank is 11/NW/0382). The current analysis was approved under UK Biobank Project 30418 and 81499. Bonafide researchers can request access to UK Biobank data via the Access Management System (AMS).

Human research participants

Policy information about studies involving human research participants and Sex and Gender in Research.

Reporting on sex and gender	Sex was considered in our analyses. Our findings of age at menarche and age at menopause relate directly to females whilst parity/number of children relate to females and males, respectively.
Population characteristics	The characteristics of these participants are shown in Table 1. At recruitment (baseline) women and men were aged (mean) 56 (SD=8.0) and 57 years (SD=8.2), 21% and 26% drank three or four times a week and 40% and 51% were previous/currents smokers, respectively. 81% of women had one or more live births whilst the mean age of menarche was 13 years (SD=1.3). 59% (37,248) women reported they went through a natural menopause with a mean age of menopause of 49.7 years (SD=5.1) [taken from results section].
Recruitment	We used data from 121,577 UK Biobank participants (54% women) with 249 metabolic measures quantified by nuclear magnetic resonance (NMR). Findings from our multivariable regression analyses for age at natural menopause should be interpreted with caution given 40% of women were excluded from these analyses as they had not experienced a natural menopause and 7% of the women had experienced menopause less than two years before study recruitment (when blood samples for NMR metabolomics were collected). In follow-up analyses, we have shown that discrepancy in findings between multivariable and MR for LDL-related traits were related to the exclusion of younger pre-menopausal women in multivariable regression [taken from results/discussion sections].
Ethics oversight	UK Biobank received ethical approval from the Research Ethics Committee (REC reference for UK Biobank is 11/NW/0382). The current analysis was approved under UK Biobank Project 30418.

Note that full information on the approval of the study protocol must also be provided in the manuscript.

Field-specific reporting

Please select the one below that is the best fit for your research. If you are not sure, read the appropriate sections before making your selection.

☒ Life sciences ☐ Behavioural & social sciences ☐ Ecological, evolutionary & environmental sciences

For a reference copy of the document with all sections, see nature.com/documents/nr-reporting-summary-flat.pdf

Life sciences study design

All studies must disclose on these points even when the disclosure is negative.

Sample size	No sample size calculation was conducted as this analysis was based on secondary data collection (UK Biobank). We used phase 1 data on metabolic traits assessed using a targeted high-throughput NMR metabolomics platform (Nightingale Health Ltd; biomarker quantification version 2020). We were provided pre-release data (now available from UKB) from a random subset of 126,846 non-fasting plasma samples collected at baseline or first repeat assessment. After removing duplicates and observations not passing quality control (QC), 121,577 samples were retained for analyses. We believe the sample size to be sufficient (121,577 UK Biobank participants (54% women)).
Data exclusions	By definition we excluded women who had not yet gone through the menopause or who had a surgical menopause in the multivariable regression. Whilst for the two sample mendelian randomisation this includes all women's genetically predicted age at menopause.
Replication	Duplicates (of those with repeated metabolite measures) were removed from our final dataset and therefore all analyses are based on independent observations.
Randomization	As this is a cohort study exploring reproductive traits we did not randomise our exposures: age at menarche, parity, and age at menopause. However, in the regression analyses, based on prior knowledge and the data, we controlled for as many confounders as possible using multivariable regression. In the main analysis we adjusted for the following confounders: adjusted for education, age at baseline and body composition at age 10. By doing so, we aimed to ensure that our estimates of interest were influenced mainly by the effects of the reproductive traits we were studying, minimizing potential bias from other factors. For the mendelian randomisation analyses, conceptually this can be thought of as a natural experiment where genetic variants instrumenting for exposures are randomly allocated at conception. Sensitivity analyses testing these assumptions were carried out. For example, in the the multivarable regression we present and compare three sets of models each adjusting for different covariates.
Blinding	As this was a cohort study investigating the associations between reproductive traits (natural exposures), namely age at menarche, parity, and age at menopause (our exposures), and various metabolic traits, blinding was not applicable. In a cohort study, researchers observe and follow a group of participants over time, collecting data on their exposure status and outcomes. In this context, the exposure information (age at menarche, parity, and age at menopause) is inherent to the participants and is not influenced or altered by the study design (other than through selection as described).

Reporting for specific materials, systems and methods

We require information from authors about some types of materials, experimental systems and methods used in many studies. Here, indicate whether each material, system or method listed is relevant to your study. If you are not sure if a list item applies to your research, read the appropriate section before selecting a response.

Materials & experimental systems

n/a	Involved in the study
☒	☐ Antibodies
☒	☐ Eukaryotic cell lines
☒	☐ Palaeontology and archaeology
☒	☐ Animals and other organisms
☒	☐ Clinical data
☒	☐ Dual use research of concern

Methods

n/a	Involved in the study
☒	☐ ChIP-seq
☒	☐ Flow cytometry
☒	☐ MRI-based neuroimaging
